# HDAC4 degradation during senescence unleashes an epigenetic program driven by AP-1/p300 at selected enhancers and super-enhancers

**DOI:** 10.1186/s13059-021-02340-z

**Published:** 2021-05-10

**Authors:** Eros Di Giorgio, Harikrishnareddy Paluvai, Emiliano Dalla, Liliana Ranzino, Alessandra Renzini, Viviana Moresi, Martina Minisini, Raffaella Picco, Claudio Brancolini

**Affiliations:** 1 Department of Medicine, Università degli Studi di Udine, p.le Kolbe 4, 33100 Udine, Italy; 2 DAHFMO Unit of Histology and Medical Embryology, Sapienza University of Rome, via Antonio Scarpa 16, 00161 Rome, Italy

**Keywords:** OIS, Senescence, Class IIa HDACs, H3K27, Super-enhancers, BRD4, H3K4me1, SASP, HDAC4, AP-1, p300

## Abstract

**Background:**

Cellular senescence is a permanent state of replicative arrest defined by a specific pattern of gene expression. The epigenome in senescent cells is sculptured in order to sustain the new transcriptional requirements, particularly at enhancers and super-enhancers. How these distal regulatory elements are dynamically modulated is not completely defined.

**Results:**

Enhancer regions are defined by the presence of H3K27 acetylation marks, which can be modulated by class IIa HDACs, as part of multi-protein complexes. Here, we explore the regulation of class IIa HDACs in different models of senescence. We find that HDAC4 is polyubiquitylated and degraded during all types of senescence and it selectively binds and monitors H3K27ac levels at specific enhancers and super-enhancers that supervise the senescent transcriptome. Frequently, these HDAC4-modulated elements are also monitored by AP-1/p300. The deletion of HDAC4 in transformed cells which have bypassed oncogene-induced senescence is coupled to the re-appearance of senescence and the execution of the AP-1/p300 epigenetic program.

**Conclusions:**

Overall, our manuscript highlights a role of HDAC4 as an epigenetic reader and controller of enhancers and super-enhancers that supervise the senescence program. More generally, we unveil an epigenetic checkpoint that has important consequences in aging and cancer.

**Supplementary Information:**

The online version contains supplementary material available at 10.1186/s13059-021-02340-z.

## Background

Cellular senescence and aging are complex responses characterized by proliferative arrest and loss of regenerative potential [1]. The senescence state is distinguished by a deep epigenetic reprogramming that sculptures the chromatin to arrest the cell cycle, maintain cellular survival, and secrete apocrine and paracrine factors [2].

Alterations in the H3K27ac/H3K27me3 ratios were identified as the driving forces of premature senescence and cancer [3–5]. The epigenetic control at distal regulatory elements is an important switch in the establishment of the senescent status [6, 7]. The histone acetyltransferase p300 promotes the formation of active enhancer elements that propel the senescence-specific gene expression program [5]. The transcription factor (TF) activator protein 1 (AP-1) is a key regulator of the transcriptional program required to launch the oncogene-induced senescence (OIS). This activity is supervised through the binding of senescence enhancers [8]. AP-1 can act as a pioneer onto the senescence enhancers to orchestrate the transcriptional program of senescent cells [8, 9].

Although some knowledges have been acquired during the last years on enhancers and super-enhancers activation in senescence, the mechanisms counteracting this activation are much less characterized. Enhancer regions are defined by characteristic histone modifications (e.g., H3K27ac and H3K4me1) [10]. The balanced action of acetyltransferases (HAT) such as p300/CBP, by interacting with the SWI/SNF complex [11] and HDACs of the Sin3, NuRD, CoREST, MiDAC, and NCOR complexes [12, 13], controls the acetylation status of H3K27. In vertebrates, class IIa HDACs are catalytically inactive epigenetic readers, quickly recruited on H3K27ac loci [14–17]. Here, they can monitor the acetylation status of H3K27 through the interaction with class I HDACs [18]. Class IIa HDACs supervise specific differentiation programs and different adaptive responses. Dysregulations of class IIa HDACs can contribute to cancer development and other diseases [18, 19]. In this manuscript, we have investigated in a comprehensive manner the regulation of class IIa HDACs during senescence and aging. Our results point to a key role of HDAC4 in counteracting replicative senescence and oncogene-induced senescence (OIS). HDAC4 by monitoring H3K27ac levels at selected enhancers and super-enhancers antagonizes the activation of the senescence gene expression program.

## Results

### HDAC4 expression is downregulated during different models of cellular senescence and with aging

**Fig. 1:**
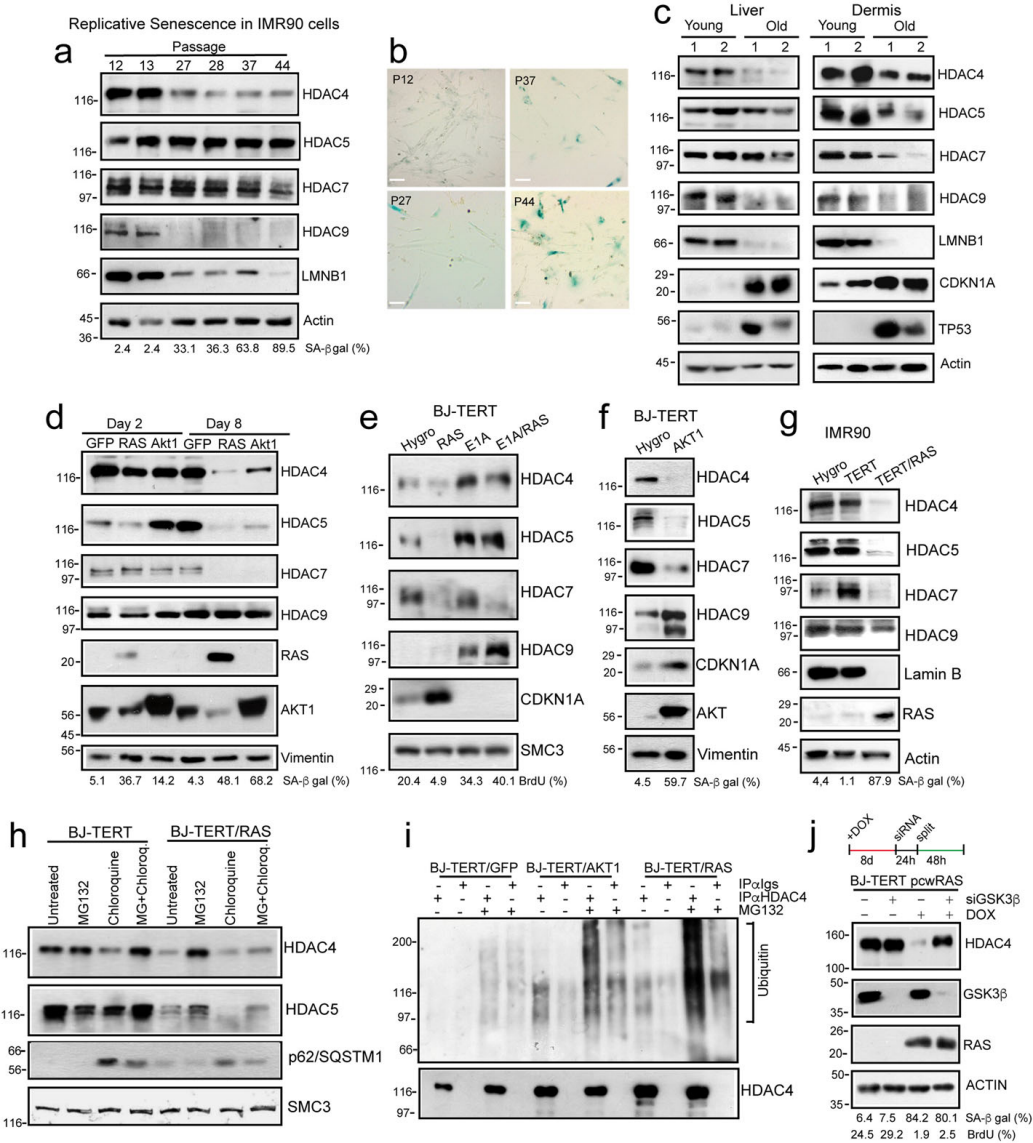
HDAC4 is dysregulated by UPS-mediated degradation during different models of senescence and aging. **a** Immunoblot analysis in IMR90 cells undergoing replicative senescence, using the indicated antibodies. The percentage of SA-β-gal positive cells is indicated. Actin was used as loading control. **b** Representative microscopic images of SA-β-gal stained IMR90 cells (scale bar 50 μm). **c** Immunoblot analysis in tissue-derived lysates obtained from C57BL/6 J female mice sacrificed at 128 (young) and 774 (old) days of age using the indicated antibodies. Actin was used as loading control. **d** Immunoblot analysis in BJ/*hTERT* cells expressing the different transgenes for the indicated time. Vimentin was the loading control. The percentage of SA-β-gal positive cells is indicated. **e** Cellular lysates were obtained from BJ/*hTERT* cells expressing the indicated transgenes. Immunoblots were performed using the indicated antibodies. The percentage of BrdU positive cells is indicated. **f** Cellular lysates were obtained from BJ/*hTERT* cells expressing AKT1 or Hygro as control. Immunoblots were performed using the indicated antibodies. The percentage of SA-β-gal positive cells is indicated. **g** Cellular lysates were obtained from IMR90 cells expressing the indicated transgenes. Immunoblots were performed using the indicated antibodies. The percentage of SA-β-gal positive cells is indicated. **h** Immunoblot analysis of HDAC4, HDAC5, and p62 in BJ/*hTERT* and BJ/*hTERT/RAS* expressing cells after 12 days of culture and 8 h of treatment with MG132 (1 μM) and chloroquine (10 μM), as indicated. SMC3 was used as loading control. **i** Cellular lysates obtained in BJ/*hTERT* expressing for 8 days the indicated transgenes and treated or not for 8 h with MG132 were immunoprecipitated using anti-HDAC4 and immunoblotted with the indicated antibodies. **j** Immunoblot analysis using the indicated antibodies in BJ/*hTERT* cells expressing HRAS^G12V^ and silenced or not for GSK3β. RAS expression was induced by DOX treatment

The epigenetic reprogramming plays essential roles in establishing cellular senescence and aging. In this context, the contribution of class IIa HDACs has been suggested [20, 21], but not addressed in a comprehensive manner. It is known that important epigenetic modulators of senescence and aging are downregulated during senescence onset [4, 22, 23]. Hence, we initially evaluated class IIa HDACs expression levels in different models of senescence and aging. HDAC4 and HDAC9, and to a lesser extent HDAC7, are progressively downregulated in human IMR90 fibroblasts undergoing replicative senescence, with a timing similar to Lamin-B1 (Fig. 1a/b). As a model of aging, we compared the levels of class IIa HDACs in the dermis and in the liver of young (4 months) and old (25 months) female mice. In humans, female longevity tends to exceed male longevity; for this reason, we investigated Hdac4 levels specifically in female mice [24]. HDAC4 and HDAC9 levels decrease, both in aged dermis and liver, whereas HDAC5 decreases only in old dermis. As expected, aged tissues are characterized by high TP53 levels and Lamin-B1 downregulation (Fig. 1c). In agreement with our data, in mice representing a senescence-accelerated model of aging, the downregulation of HDAC4 was previously observed [25]. Therefore, telomere attrition in normal cells and physiological aging, similarly affect HDAC4 and HDAC9 protein levels.

Next, we used additional models of cellular senescence to investigate the behavior of class IIa HDACs: oncogene-induced senescence (OIS) [26, 27], stress-induced senescence (SISP), following H_2_O_2_ treatment, and cytokine-induced senescence [28]. The induction of senescence was verified by classical markers (SA-β gal staining positivity, BrdU negativity, CDKN1A upregulation and LMNB1 downregulation). The delivery in BJ/*hTERT* cells of the strong oncogenes HRAS^G12V^ (thereafter RAS) or myrAKT1 triggers OIS (Additional file 1: Fig. S1a). In these inducible models of OIS, the downregulation of HDAC4, HDAC5, and HDAC7 occurs after the initial proliferative phase, at day 8 from oncogene induction (Fig. 1d). Class IIa HDACs downregulation is coupled to the upregulation of *CDKN1A*, *CDKN2A*, and *GADD45A* transcription (Additional file 1: Fig. S1b).

Oncogenes of viral origin, like E1A and its ΔC-fragment (1-143), are able to overcome OIS, by targeting the p16/Rb pathways [29]. HDAC4/5/9, but not HDAC7, are upregulated by the expression of E1AΔC in BJ/*hTERT* cells (Fig. 1e). Moreover, the co-expression of E1A and RAS bypasses the senescent arrest and recovers the protein levels of HDAC4 and HDAC5, but not of HDAC7 (Fig. 1e). The same regulation of class IIa HDACs was confirmed in a second model of AKT1-induced OIS (Fig. 1f) and in IMR90 fibroblasts during RAS-induced OIS (Fig. 1g). Curiously, RAS triggers a reduction of HDAC9 levels in IMR90 cells compared to BJ cells. The higher percentage of SA-β-gal positive cells suggests that IMR90/TERT/RAS cells are in a more advanced state of senescence compared to BJ/TERT/RAS cells. HDAC4 and HDAC5 are decreased also during SISP and cytokine-induced senescence (Additional file 1: Fig. S1c/d). In summary, our screening identified HDAC4 as the class IIa HDAC member repressed in all tested models of senescence and in aging.

Senescence marginally affects class IIa HDACs mRNA levels. The exception was HDAC9, whose mRNA increases in response to oncogene activation (Additional file 1: Fig. S1e/f/g). Therefore, the downregulation of HDAC4 during OIS could be mediated by the ubiquitin-proteasome system (UPS) as demonstrated in other contexts [30]. During OIS, HDAC4 levels are restored by the proteasome inhibitor MG132 but not after autophagy inhibition (Fig. 1h). Curiously, autophagy blockage suppresses HDAC4 expression independently from OIS. Accordingly, HDAC4 is highly poly-ubiquitylated in senescent cells (Fig. 1i and Additional file 1: Fig. S1h). Previous studies have shown that the ubiquitin-dependent degradation of HDAC4 depends on GSK3β [30]. Similarly, in senescent cells, the treatment with LiCl (Additional file 1: Fig. S1i) and the silencing of GSK3β (Fig. 1j) are both capable of restoring HDAC4 levels.

Class IIa HDACs nuclear/cytoplasmic shuttling is intensively regulated by phosphorylation. We investigated the levels of serines 246/259 phosphorylation, found respectively in HDAC4 and HDAC5, during RAS-induced senescence (RIS). These serines, that are binding sites for 14-3-3 proteins and involved in the nuclear export, are rapidly dephosphorylated upon RAS induction (Additional file 1: Fig. S1j). However, HDAC4 shows a pan/nuclear localization in BJ-TERT cells, and this localization is largely unperturbed after RAS activation (Additional file 1: Fig. S1k). In conclusion, dephosphorylation at serines 246/259 is an early response to RAS induction, coupled to the proliferative boost.

### The depletion of HDAC4 anticipates senescence while its re-expression delays RAS-induced senescence (RIS)

We established different cellular models to investigate the role played by HDAC4 in regulating senescence onset and escape. Firstly, we conditionally knocked-out *Hdac4* (4-OHT dependent) in primary murine embryonic fibroblasts (MEFs). MEFs undergo senescence rapidly when grown at atmospheric oxygen, while the maintenance of hypoxic conditions preserves their proliferation [31]. The conditional KO of *Hdac4* in MEFs under normoxia anticipates the progressive increase in Cdkn2a/p16 levels (Additional file 1: Fig. S2a). Accordingly, *Hdac4*^*−/−*^ MEFs arrest the proliferation earlier (Additional file 1: Fig. S2b/c) and anticipate the upregulation of genes related to senescence such as *Cdkn2a*, *Il1b*, *Ifnb*, and *irf7* (Additional file 1: Fig. S2d). As expected, when MEFs are grown in hypoxic conditions senescence is not observed (Additional file 1: Fig. S2e/f). However, prior to immortalization, *Hdac4*^*−/−*^ MEFs experience a proliferative crisis between 40 and 50 days of culture (Additional file 1: Fig. S2f).

**Fig. 2:**
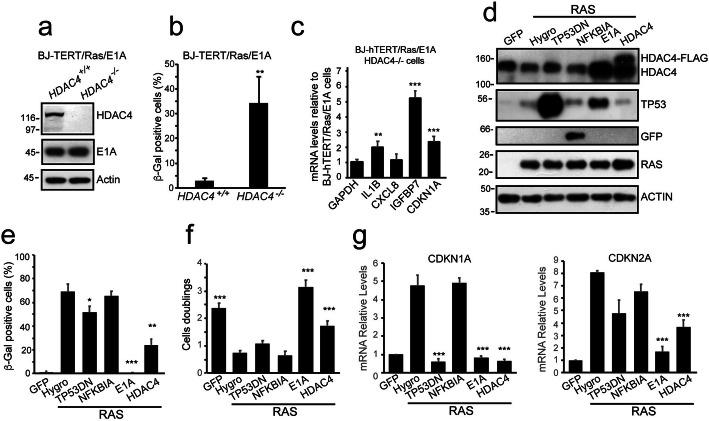
HDAC4 is required for senescence escape. **a** Immunoblot analysis in BJ/*hTERT/E1A/RAS*/*HDAC4*^*+/+*^ or ^*−/−*^ cells, as indicated. Actin was used as loading control. **b** Analysis of the senescent cells as scored after SA-β-gal staining. Mean ± SD; *n* = 3. **c** RNA-expression levels of the indicated genes in BJ/*hTERT/E1A/RAS*/*HDAC4*^*−/−*^ with respect to WT cells. Mean ± SD; *n* = 3. **d** Immunoblot analysis in BJ/*hTERT* cells expressing the indicated transgenes. Where indicated, *HRAS*^*G12V*^ was induced for 9 days. Anti-GFP was used to visualize GFP-NFKBIA. Actin was used as loading control. **e** Analysis of the senescence as scored after SA-β-gal staining in BJ/*hTERT* cells expressing the indicated transgenes. **f** Analysis of the population doublings in BJ/*hTERT* cells expressing the indicated transgenes, scored during the 9 days of observation. **g** RNA-expression levels of the indicated genes in the cells co-expressing RAS and the indicated transgenes, with respect to HYGRO expressing cells not expressing RAS. Mean ± SD; *n* = 3. The significance is relative to RAS+Hygro^R^ co-expressing cells

Secondly, we tested the role played by HDAC4 in regulating OIS escape in human cells. The BJ/*hTERT/Ras/E1A* cells are a classical model of OIS escape [29]. CRISPR-mediated HDAC4 KO (Fig. 2a and Additional file 1: Fig. S3a/b) in BJ/*hTERT/Ras/E1A* cells causes the appearance of SA-β-gal positive cells (Fig. 2b) and triggers the expression of senescence-related genes (Fig. 2c). The induction of senescence in these cells is unrelated to E1A levels (Fig. 2a).

To further characterize HDAC4 as an important element in counteracting OIS, we evaluated its ability to reverse OIS upon RAS induction. The effect of HDAC4 was compared to the silencing of pRB, TP53, or NFKB pathways, well-known regulators of the senescence response. They are involved in the control of the cell cycle (pRB), in sensing oncogenic lesions/DNA damage (TP53), and in SASP surveillance (NFKB) [2, 28]. E1A, the TP53DN dominant negative mutant (TP53^R175H^) [32], the NFKB inhibitor IKBα S32A/S36A [33], referred hereinafter as NFKBIA, and HDAC4 were over-expressed in BJ/h*TERT* cells induced to express RAS for 9 days to reach OIS. Increasing the levels of HDAC4 in BJ/h*TERT/RAS*-ER cells decreases senescence onset (Fig. 2e) and sustains cell proliferation (Fig. 2f). HDAC4 also represses *CDKN1A* and *CDKN2A* expression (Fig. 2g). Only E1A shows a stronger OIS bypass activity compared to HDAC4.

### Depletion of HDAC4 triggers senescence in cancer cells

**Fig. 3:**
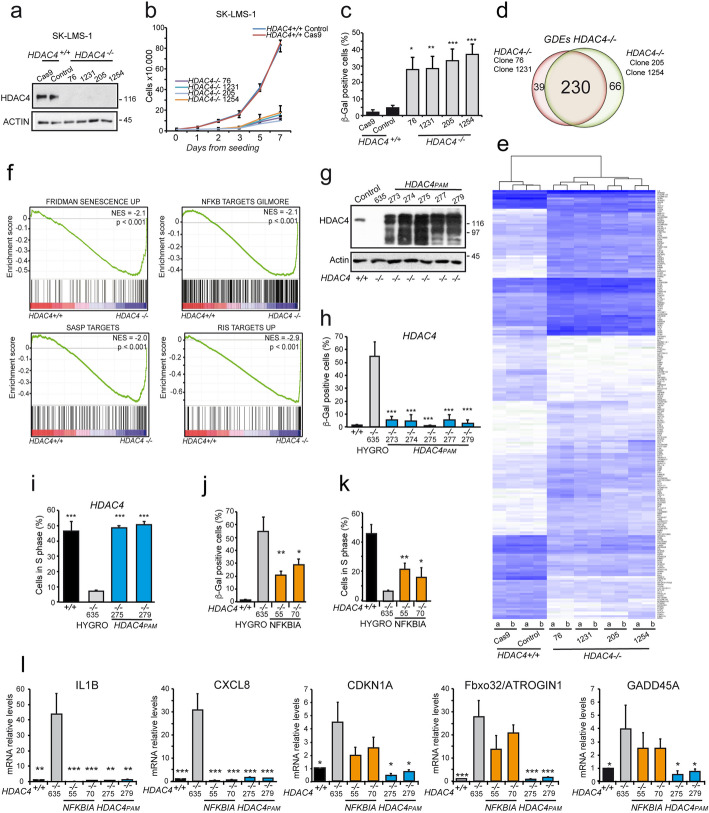
Depletion of HDAC4 triggers senescence in cancer cells. **a** Immunoblot analysis in SK-LMS-1/*HDAC4*^*+/+*^ or *HDAC4*^*−/−*^ cells as indicated. Actin was used as loading control. **b** Cell-proliferation curve of the indicated *HDAC4*^*+/+*^ and *HDAC4* ^*−/−*^ SK-LMS-1 cells. Mean ± SD; *n* = 4. **c** Analysis of the senescent cells as scored after SA-β-gal staining. Mean ± SD; *n* = 4. **d** Venn diagrams showing the number of transcripts differently regulated in SK-LMS-1/*HDAC4*^*−/−*^ cells generated by using two different sgRNAs (green and red). Differentially expressed genes (DEGs) were selected based on |fold change| > 2 and *p* < 0.05. **e** Heat-map of the absolute expression levels of the DEGs in the indicated clones and biological replicates hierarchically clustered accordingly to average linkage. Blue shades intensity is proportional to transcripts abundance. **f** GSEA plots displaying the NES obtained by interrogating the transcriptome of HDAC4 WT and KO cells with the indicated gene sets. **g** Immunoblot analysis in SK-LMS-1 *HDAC4*^*+/+*^ or *HDAC4*^*−/−*^, stably re-expressing *Hygro*^*R*^ (clone 635) or HDAC4 (clones 275 and 279), as indicated. **h** Analysis of SA-β-gal positivity in WT or *HDAC4*^*−/−*^ SK-LMS-1 cells expressing *HYGRO*^*R*^ (clone 635) or *HDAC4* (clones 273–279). Mean ± SD; *n* = 4. The significance is relative to the KO clone 635. **i.** Analysis of BrdU incorporation in WT or *HDAC4*^*−/−*^ SK-LMS-1 cells expressing *HYGRO*^*R*^ (clone 635) or *HDAC4* (clones 273–279). Mean ± SD; *n* = 4. The significance is relative to the KO clone 635. **j** Analysis of SA-β-gal positivity in SK-LMS-1 WT or *HDAC4* KO cells expressing *HYGRO*^*R*^ (clone 635) or *GFP-NFKBIA* (clones 55 and 70). Mean ± SD; *n* = 4. **k** Analysis of BrdU incorporation in SK-LMS-1 WT or *HDAC4* KO cells expressing *HYGRO*^*R*^ (clone 635) or *GFP-NFKBIA* (clones 55 and 70). Mean ± SD; *n* = 4. **l** mRNA expression levels of the indicated genes in the indicated SK-LMS-1 WT or *HDAC4*^*−/−*^ clones re-expressing *HYGRO*^*R*^ (clone 635), *HDAC4* (clones 275–279) or *GFP-NFKBIA* (clones 55–70). Mean ± SD; *n* = 3. The significance is relative to the KO clone 635

Since class IIa HDACs are abundantly expressed in leiomyosarcomas (LMS) [15, 34], to confirm the role of HDAC4 in senescence escape, its expression was knocked-out also in low grade LMS cells SK-LMS-1 [35] (Fig. 3a and Additional file 1: Fig. S3a/b). In this additional model of OIS escape, HDAC4 depletion arrests proliferation (Fig. 3b) and induces SA-β-gal positivity (Fig. 3c and Additional file 1: S3c). All KO clones fail to grow in semisolid medium (Additional file 1: Fig. S3d/e). These phenotypes are reproducible in the 4 KO clones generated with 2 different sgRNAs (sg1: 76, 1231; sg2: 205, 1254). Removal of HDAC4 also mildly increases the level of apoptosis (Additional file 1: Fig. S3f).

To better characterize this senescent program, the transcriptomes of four LMS *HDAC4*^*−/−*^ clones were compared with two control clones (expressing Cas9 or Cas9/sgRNA1 but in which the KO was not achieved). The principal-component analysis is shown in Fig. S3g (Additional file 1). By applying stringent statistical criteria, we identified a minimal signature of 230 genes significantly modulated in all *HDAC4*^*−/−*^ clones (Fig. 3d). One hundred forty-two out of 230 of these genes (62%) are induced after HDAC4 deletion (Fig. 3e). An unbiased GSEA analysis identified a senescence geneset among the most enriched in *HDAC4*^*−/−*^ cells (Fig. 3f). Moreover, senescence-associated secretory phenotype (SASP), RIS, and NFKB1 target genes are all positively enriched in *HDAC4*^*−/−*^ cells (Fig. 3f).

To unambiguously prove that the CRISPR-mediated knock-out of HDAC4 is the primary cause of senescence, we re-expressed a Cas9 resistant mutant of HDAC4 (*HDAC4*^*PAM*^) (Fig. 3g). This expression rescues the senescent phenotype in all clones (Fig. 3h), stimulates the entering into the cell cycle (Fig. 3i), and supports their growth in soft agar (Additional file 1: Fig. S3h). As expected, NFKB inhibition only partially recovers the senescent phenotype in terms of SA-β-gal positivity and progression through S-phase (Fig. 3j/k) [36].

Finally, the re-expression of HDAC4 in SK-LMS-1/*HDAC4*^*−/−*^ cells rescues the expression of a set of genes, which define the SASP and the cell cycle arrest. Differently, blocking NFKB affects only the SASP genes and not the cell cycle related genes (Fig. 3l).

As the last model of senescence, we knocked-out HDAC4 in A375 melanoma cells. Melanoma was chosen since senescence barrier disruption during melanomagenesis is well reported [37]. Moreover, high levels of HDAC4 negatively correlate with patients’ survival (Additional file 1: Fig. S4a). Initially, we obtained only heterozygous clones, suggesting an important role of HDAC4 in regulating cell fitness. Therefore, we generated (Doxycyline) DOX-inducible cell lines to conditionally express *HDAC4*^*PAM*^ before its targeting. With this strategy, five *HDAC4*^*−/−*^ (320, 304, 401, 150, 1090) and two *HDAC4*^*+/−*^ clones (159, 317) were isolated (Additional file 1: Fig. S4b). Similarly to LMS cells, A375/*HDAC4*^*−/−*^ cells evidence the downregulation of LMNB1 (Additional file 1: Fig. S4b/f) and the acquisition of classical senescent markers like cell cycle arrest (Additional file 1: Fig. S4c), SA-β-gal positivity (Additional file 1: Fig. S4d), and the appearance of cytosolic chromatin fragments (Additional file 1: Fig. S4e). All these phenotypes are weaker in the heterozygous (*HDAC4*^*+/−*^) clones and the re-expression of HDAC4 recovers the normal phenotype. The above-described genes repressed by HDAC4 in LMS cells turned out to be upregulated also in A375 *HDAC4*^*−/−*^ cells (Additional file 1: Fig. S4g). The role of HDAC4 in antagonizing senescence in melanomas was confirmed by RNAi mediated silencing in WM115 cells (Additional file 1: Fig. S4h). LMNB1 downregulation, TP53 upregulation (Additional file 1: Fig. S4h), increased SA-β-gal positivity (Additional file 1: Fig. S4i), and the upregulation of a pool of senescent-related genes regulated by HDAC4 in LMS cells (Additional file 1: Fig. S4J) were all observed after HDAC4 silencing.

### The epigenome of *HDAC4*^*−/−*^ cells is altered at the level of specific loci

HDAC4 is an epigenetic regulator that supervises H3K27ac levels, particularly at distal regulatory regions [15, 38]. We hypothesized that HDAC4 could safeguard the epigenetic identity to counteract senescence. Having proved the role of HDAC4 in different models of OIS, we decided to investigate more in detail the epigenetic changes in LMS cells. This choice is justified by the following: (i) the role of class IIa and of their partners in this tumor is well characterized [15, 34, 39] and (ii) mutations in two key elements of the senescent pathway, *RB1* and *CDKN2A*, in LMS are mutually exclusive and strongly associate with patient survival (Fig. S5a-b). To evaluate the epigenomic influence of HDAC4 depletion during the initial steps of senescence, we generated SK-LMS-1 *HDAC4*^*−/−*^ cells that re-express a 4-OHT inducible HDAC4, *HDAC4*^*−/−*^*/HDAC4*^*PAM*^*-ER* (Additional file 1: Fig. S5c). The tamoxifen inducible re-expression of *HDAC4* in SK-LMS-1/*HDAC4*^*−/−*^ cells (clone 66, + 4-OHT) impairs the appearance of senescence, which can be quickly established following removal of 4-OHT. This effect was proved by the abrogation of SA-β-gal positivity (Additional file 1: Fig. S5d), by the restoration of LMNB1 levels (Additional file 1: Fig. S5c) and cell proliferation (Additional file 1: Fig. S5e) and by the growth in soft-agar (Additional file 1: Fig. S5f). Moreover, the re-expression of HDAC4 abrogates two typical features of senescent cells: the sensitivity to the senolytic drug navitoclax/ABT-263 [40] (Additional file 1: Fig. S5g) and the loss of the linker histone H1 [41] (Additional file 1: Fig. S5h).

**Fig. 4:**
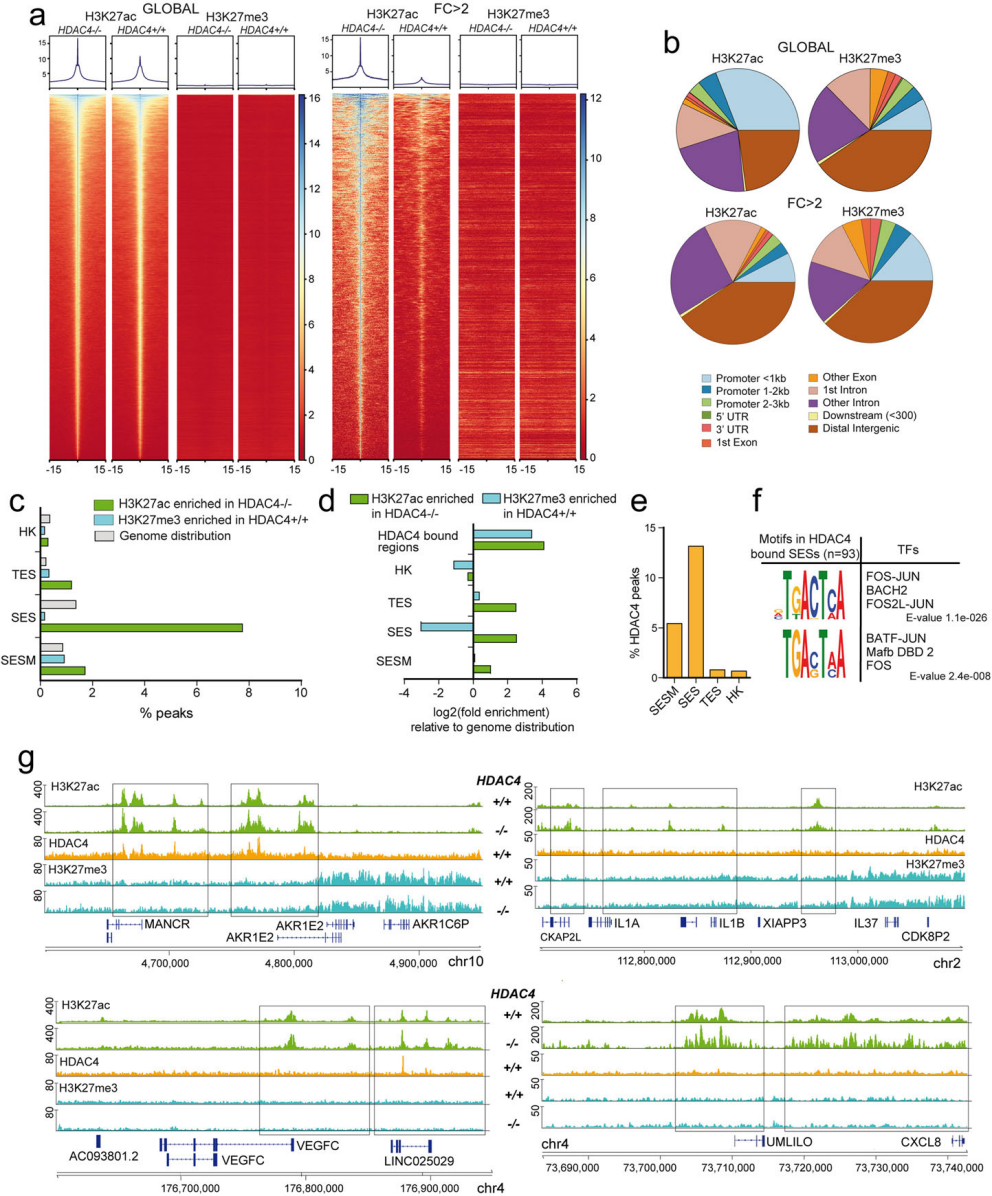
HDAC4 supervises enhancers and super-enhancers activated early during senescence. **a** (Left, marked as “GLOBAL”) Heat-map of the 97,831 H3K27ac and the corresponding H3K27me3 signals in the indicated SK-LMS-1 cells; (right, marked as “FC > 2”) heat-map of the 4441 H3K27ac peaks displaying an overall signal ratio (KO/wt) > 2 and the corresponding H3K27me3 signals in the indicated SK-LMS-1 cells. The analysis was performed in a region of ± 15Kb around peak summit. **b** Genomic distribution of all the H3K27ac and H3K27me3-enriched peaks (top panel), or only of those associated with a FC > 2, as indicated and shown in **a**. **c** Histogram representing the percentage of hyper-acetylated (green bar) or demethylated (light blue) H3K27 peaks in SK-LMS-1/*HDAC4*^*−/−*^ cells and falling in the indicated genomic elements or displaying the indicated epigenomic features. The genome coverage of each element is indicated by gray bars. **d** Histogram representing the enrichment of each element described in Fig. 4c with respect to the expected distribution calculated according to the genome coverage. **e** Histogram representing the percentage of HDAC4 peaks falling in WT cells in correspondence to the indicated genomic elements or displaying the indicated epigenomic features in *HDAC4*^*−/−*^ cells. **f** Motif analysis of 91 HDAC4-gained SES for putative transcription factor binding sites. Motifs with *p* value< 0.5 × 10^−4^ were selected. **g** (Left) Detailed view of 2 representative SES (*AKR1E2* and *VEGFC* associated SES) directly bound by HDAC4 in SK-LMS-1 WT cells in correspondence to H3K27ac-defined SES activated after HDAC4 depletion. (Right) Detailed view of 2 representative SES (*IL1B* and *CXCL8* associated SES) not directly bound by HDAC4 in SK-LMS-1 WT cells, but found activated after HDAC4 depletion

Next, ChIP-seq was performed in *HDAC4*^*−/−*^*HDAC4*^*PAM*^*-ER* cells at 36 h from the removal of 4-OHT, to monitor H3K27ac and H3K27me3 variations. Genome-wide analysis shows locus specific increases of H3K27ac levels in cells knocked-out for HDAC4. These loci do not show overt variations in H3K27me3 (Fig. 4a/b). In parallel, HDAC4 depletion triggers moderate decreases of H3K27me3 levels in loci that do not show overt variations in H3K27 acetylation (Additional file 1: Fig. S6a/b). The re-acetylation observed in *HDAC4*^*−/−*^ cells involved mainly intronic (18%) and intergenic regions (45%), as similarly observed in other studies [15–17]. Finally, the reasons for H3K27me3 increase in some loci remain to be tested.

The enrichment for epigenetic modifications in intergenic regions upon HDAC4 depletion leads us to hypothesize that HDAC4 could monitor distal regulatory elements such as typical enhancers (TE) and super-enhancers (SE) associated with senescence. SE are large clusters of TE that are densely occupied by transcription factors and co-regulators of transcription [42]. Hence, we defined an atlas of activated TE and SE that characterize cellular senescence by joining H3K27ac peaks enriched during replicative senescence [5] and OIS [4] (Additional file 2, Table S1). We defined them as TES (typical enhancers of senescence) and SES (super-enhancers of senescence), in respect to SESM (super-enhancers of smooth muscles) that typify this cell lineage. Two percent and 8% of the H3K27ac regions hyper-acetylated in *HDAC4*^*−/−*^ cells are found respectively in TES and SES (Fig. 4c).

### HDAC4 supervises enhancers and super-enhancers activated early during senescence

The previous analysis suggests that hyper-acetylated H3K27 regions in *HDAC4*^*−/−*^ cells are enriched for TES and SES. Hence, we hypothesized that HDAC4 could directly control H3K27ac levels at TES and SES. To prove it, we used ChIP-seq to map the genomic regions bound by HDAC4. Figure 4d shows that HDAC4 binding in WT cells is enriched in correspondence of loci characterized by H3K27ac hyper-acetylation and H3K27me3 de-methylation after HDAC4 KO. This evidence suggests a direct involvement of HDAC4 in these epigenetic regulations, as further confirmed by the heatmap analysis for H3K27ac at SES (Additional file 1: Fig. S6c). In summary, 13.5% of HDAC4 peaks colocalize with SES (Fig. 4e). HDAC4-bounded chromatin regions, which organize in SES after HDAC4 depletion, are characterized by a strong consensus for Activator Protein 1 (AP-1) (Fig. 4f). Figure 4g exemplifies 4 different loci containing SES, which H3K27ac levels increase after *HDAC4* deletion. *AKR1E2* and *VEGFC* are two loci containing the AP-1 consensus and characterized by HDAC4 recruitment (Fig. 4g left panel). By contrast, NFKB consensus characterizes SES not directly bound by HDAC4 (Fig. 4g right panel and Additional file 3, Additional file 3: Table S2), as exemplified by the *IL1B* and *CXCL8* loci. The quantitative analysis confirmed an augmentation in H3K27 acetylation at these SES upon HDAC4 depletion (Additional file 4: Table S3). The activation of the TE that controls *CDKN1A* expression in HDAC4 KO cells is shown in Additional file 1: Fig. S6d.

### HDAC4 is required to load a repressive complex in correspondence to well-defined AP-1 regulated SES and antagonizes p300

**Fig. 5:**
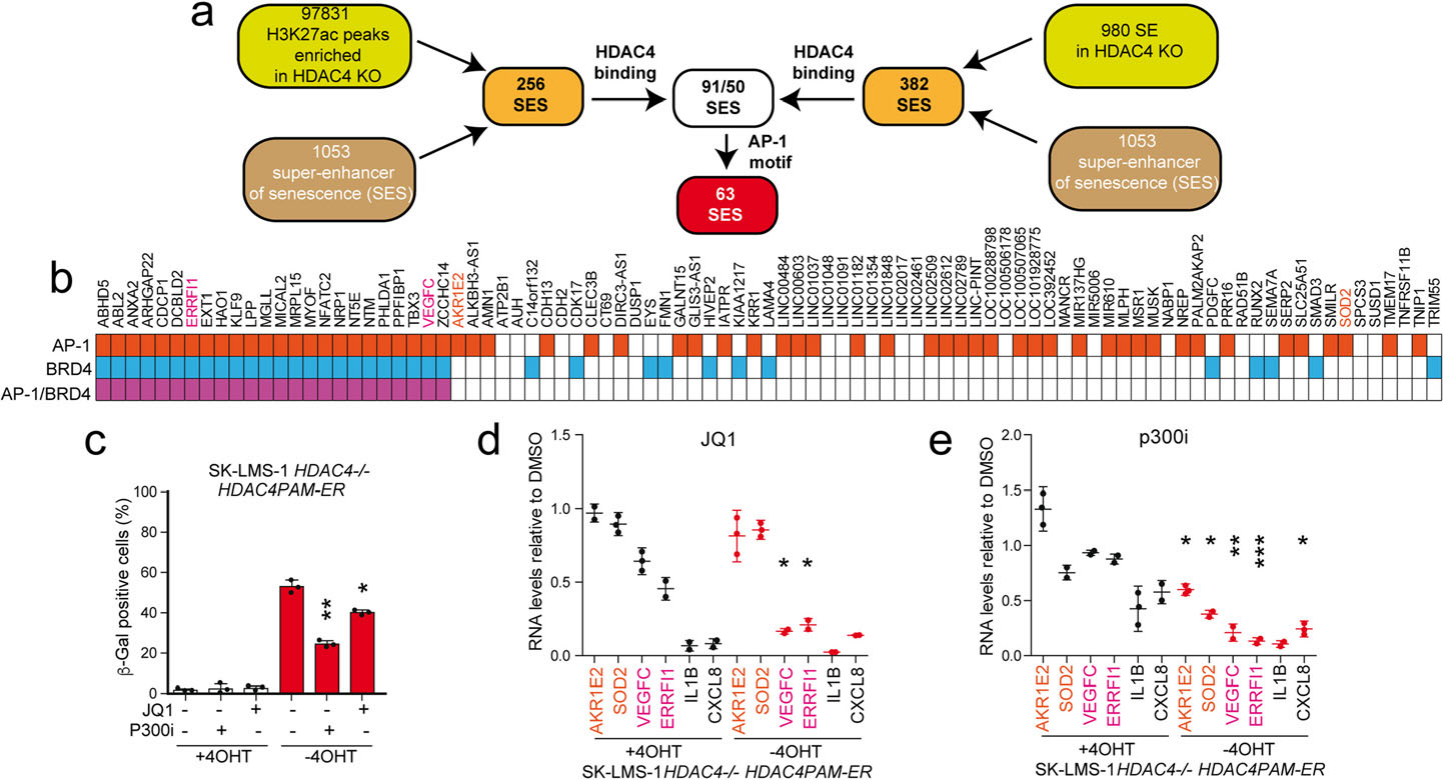
Several SES bound and regulated by HDAC4 are targets of AP-1 and p300. **a** The flow chart summarizes the screening procedure adopted to evaluate SE and TE regulated by HDAC4 and characterized for AP-1 binding motifs in senescence. **b** 91 HDAC4-bound and regulated SES were ordered according to the reported binding by AP-1 [9] (red squares), BRD4 [4] (blue squares), or both (violet squares) during senescence. *AKR1E2* and *SOD2* (in red) and *VEGFC* and *ERRFI1* (violet) were selected respectively as representative BRD4-independent and BRD4-dependent AP-1 and HDAC4 regulated SES. **c** Analysis of SA-β-gal positivity in SK-LMS-1 *HDAC4*^*−/−*^ cells, re-expressing (+ 4-OHT) or not (− 4-OHT) HDAC4-ER for 24 h prior to the treatment for 48 h with JQ-1 (0.5 μM) or 3 μM of A-485 (p300i), as indicated. Mean ± SD; *n* = 3. The significance is relative to untreated cells not re-expressing HDAC4. **d** mRNA expression levels of the indicated genes after treatment with JQ-1, as explained in **c**. For the two groups of KO cells re-expressing or not HDAC4, the fold induction is relative to the matched DMSO-treated cells. The significance is relative to the comparison between the two groups re-expressing or not HDAC4 and treated with the same drug. **e** mRNA expression levels of the indicated genes after treatment with p300i, as explained in **c**. For the two groups of KO cells re-expressing or not HDAC4, the fold induction is relative to the matched DMSO-treated cells. The significance is relative to the comparison between the two groups re-expressing or not HDAC4 and treated with the same drug

H3K27ac peaks modulated by HDAC4 can be found in 256 SES defined in other cell lines. Among them, 91 SES are directly regulated by HDAC4 and 63 of them bear the AP-1 consensus in correspondence to HDAC4 binding sites (Fig. 5a). Since SE can be cell type specific, to strengthen the importance of HDAC4 in the regulation of SES, ChIP-seq experiments were performed to map H3K4me1 distribution in SK-LMS-1 WT cells or at 36 h from HDAC4 depletion. Using the ROSE algorithm, 980 SE were globally identified in HDAC4^*−/−*^ cells (Additional file 5: Table S4). Thirty-nine percent of them were previously defined as SES in other cell lineages (Fig. 5a), and 363 were specific of HDAC4^*−/−*^ cells. This group of SE represents the SE induced during the early phases of senescence (36 h from HDAC4 depletion) in SK-LMS-1 cells. Twenty-seven of these SES are marked by HDAC4 binding. The heat maps and the metaplots for H3K27ac levels in these SES are illustrated in the Additional file 1: Fig. S6e. Relying on the importance of HDAC4 in controlling SES dynamics, we next explored the role of AP-1 in the modulation of SES under HDAC4 influence. We focused the analysis on the 63 SES that bear the AP-1 binding sites and show variations in H3K27 acetylation. One third of these AP-1 regulated loci were found to be bound by BRD4 during senescence [4] (Fig. 5b). Together with BRD4, the HAT p300 sustains most of the transcriptional activities of AP-1 [43]. BRD4 is a mediator of transcription that densely occupies SE to drive gene transcription [4]. Hence, we investigated the contribution of BRD4 and p300 in transcribing SES associated genes in *HDAC4*^*−/−*^ cells. The treatment with the p300 inhibitor (p300i/A-485) better counteracts senescence in HDAC4-depleted cells, with respect to the BRD4 inhibitor JQ-1 (Fig. 5c). We confirmed the efficiency of the inhibitors by scoring the expression of 6 SES-associated genes, 4 directly regulated (*ERRFI1*, *AKR1E2*, *SOD2*, *VEGFC*) and 2 indirectly regulated by HDAC4 (*ILB*, *CXCL8*). Of these genes, 4 were predicted to be BRD4 regulated: *VEGFC*, *ERRFI1*, *IL1B*, and *CXCL8* (Fig. 5b). As expected, JQ-1 reduces the expression of BRD4 regulated genes (*VEGFC*, *ERRFI1*, *IL1B*, *CXCL8*), both in WT and in HDAC4-depleted cells, much potently for *IL1B* and *CXCL8* (Fig. 5d). The effect is stronger in KO cells only for *VEGFC* and *ERRFI1*. Differently from JQ-1, A-485 less efficiently impacts on the transcription of the six genes in WT cells (only *IL1B* and *CXCL8*), while it strongly blunts their expression in HDAC4-depleted cells (Fig. 5e). Collectively, these results suggest that HDAC4 sculpts the chromatin by competing with p300. Its absence could favor the organization of the chromatin into super-enhancers at AP-1 binding sites.

### HDAC4 supervises the deacetylation of AP-1 regulated SES

HDAC4 assembles in multiprotein complexes where the recruitment of other co-repressors is critical to mediate its epigenetic influences. To gain insight on the mechanisms operated by HDAC4 to monitor TE and SE associated with senescence, we used the wt HDAC4 in comparison to the mutant HDAC4^Δ600^-GFP, lacking the deacetylase domain (DAC). This domain is required to recruit HDAC3 [44]. We also introduced HDAC3-GFP in HDAC4 depleted cells, for comparison.

**Fig. 6:**
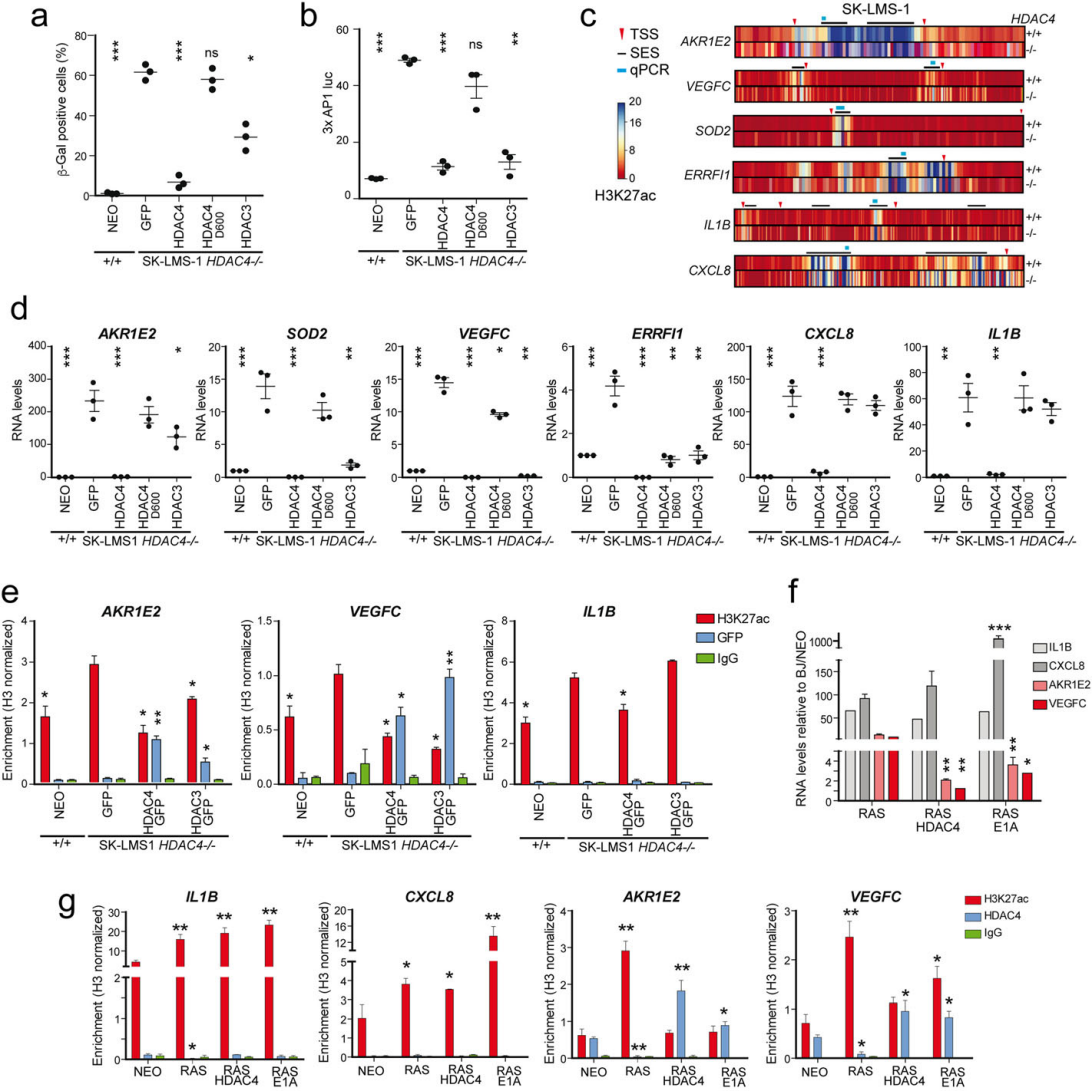
HDAC4 is required to load a repressive complex in correspondence to well-defined AP-1 regulated SES that antagonizes p300. **a** Analysis of SA-β-gal positivity in SK-LMS-1 WT cells expressing *Neo*^*R*^ and in SK-LMS-1/*HDAC4*^*−/−*^ cells re-expressing the indicated transgenes. *n* = 3. The significance is relative to KO cells expressing *Neo*^*R*^. **b** AP-1 transcriptional activity in SK-LMS-1 WT cells expressing *Neo*^*R*^ and in SK-LMS-1/*HDAC4*^*−/−*^ cells re-expressing the indicated transgenes. *n* = 3. The significance is relative to KO cells expressing *Neo*^*R*^. **c** Heat-map of the H3K27ac levels in WT and *HDAC4−/−* cells in a window of 40 kb around the analyzed SES. The TSS, the SES, and the regions analyzed by ChIP-qPCR are indicated. **d** mRNA expression levels of the indicated genes in SK-LMS-1 WT cells expressing *Neo*^*R*^ and in SK-LMS-1/*HDAC4*^*−/−*^ cells re-expressing the indicated transgenes. *n* = 3. The significance is relative to KO cells expressing Neo^R^. **e** ChIP-qPCR signals, normalized to total H3, obtained for the indicated antibodies in the indicated cells. Mean ± SD; *n* = 3. The significance is relative to KO cells expressing Neo^R^. **f** mRNA expression levels of the indicated genes in BJ/*hTERT/RAS* cells re-expressing or not HDAC4 and E1A, as indicated, in respect to BJ/*hTERT/Neo*^*R*^ cells. The significance is relative to BJ/*hTERT/Neo*^*R*^ cells. **g** ChIP-qPCR signals for the indicated loci in BJ/*hTERT/RAS* cells re-expressing or not HDAC4 and E1A, as indicated, in respect to BJ/*hTERT/Neo*^*R*^ cells. The significance is relative to BJ/*hTERT/RAS* cells

While the re-expression of full-length HDAC4-GFP completely rescues the proliferative deficit observed in HDAC4 depleted SK-LMS-1 cells, the re-expression of the mutant HDAC4^Δ600^-GFP fails in escaping senescence (Fig. 6a). A partial recovery of senescence was observed when HDAC3 is introduced (Fig. 6a). This rescue is inversely correlated with AP-1 transcriptional activity, which is deeply triggered in HDAC4 depleted cells. AP-1 transcriptional activity is not affected after GFP or HDAC4^Δ600^-GFP, but it is strongly impaired after HDAC4-GFP and, to a lesser extent, after HDAC3-GFP expression (Fig. 6b). These results find confirmation in the HDAC4-dependent levels of H3K27ac at AP-1 regulated SES-associated genes (Fig. 6c) and in the relative mRNAs levels (Fig. 6d). Particularly, the repression of SES-associated genes after HDAC3 over-expression is not successful for those loci, like *AKR1E2*, in which HDAC4 is required for the efficient loading of HDAC3 on the chromatin (Fig. 6e). Interestingly, both HDAC4-GFP and HDAC3-GFP fail to bind the SE associated with *IL1B* (Fig. 6e). Similarly, the expression of HDAC4 or E1A in BJ/h*TERT*/*RAS* cells blunts the upregulation of those SES-associated genes (Fig. 6f) that are directly bound and regulated by HDAC4 (Fig. 6g). Again, *IL1B* and *CXCL8* do not evidence any direct binding of HDAC4 and, more importantly, they are not repressed by HDAC4 or E1A, as a consequence of OIS escape (Fig. 6f). When normalization was calculated respect to input the results were identical, thus indicating that, within the time-frame of the experiment, H3 levels remain unperturbed. To summarize, HDAC4 controls the HDAC3-assisted deacetylation of a pool of AP-1-regulated SES whose de-repression is associated with senescence entrance and whose repression correlates with senescence escape.

### A local chromatin environment and not the binding qualities affects HDAC4 activity on well-defined SES

**Fig. 7:**
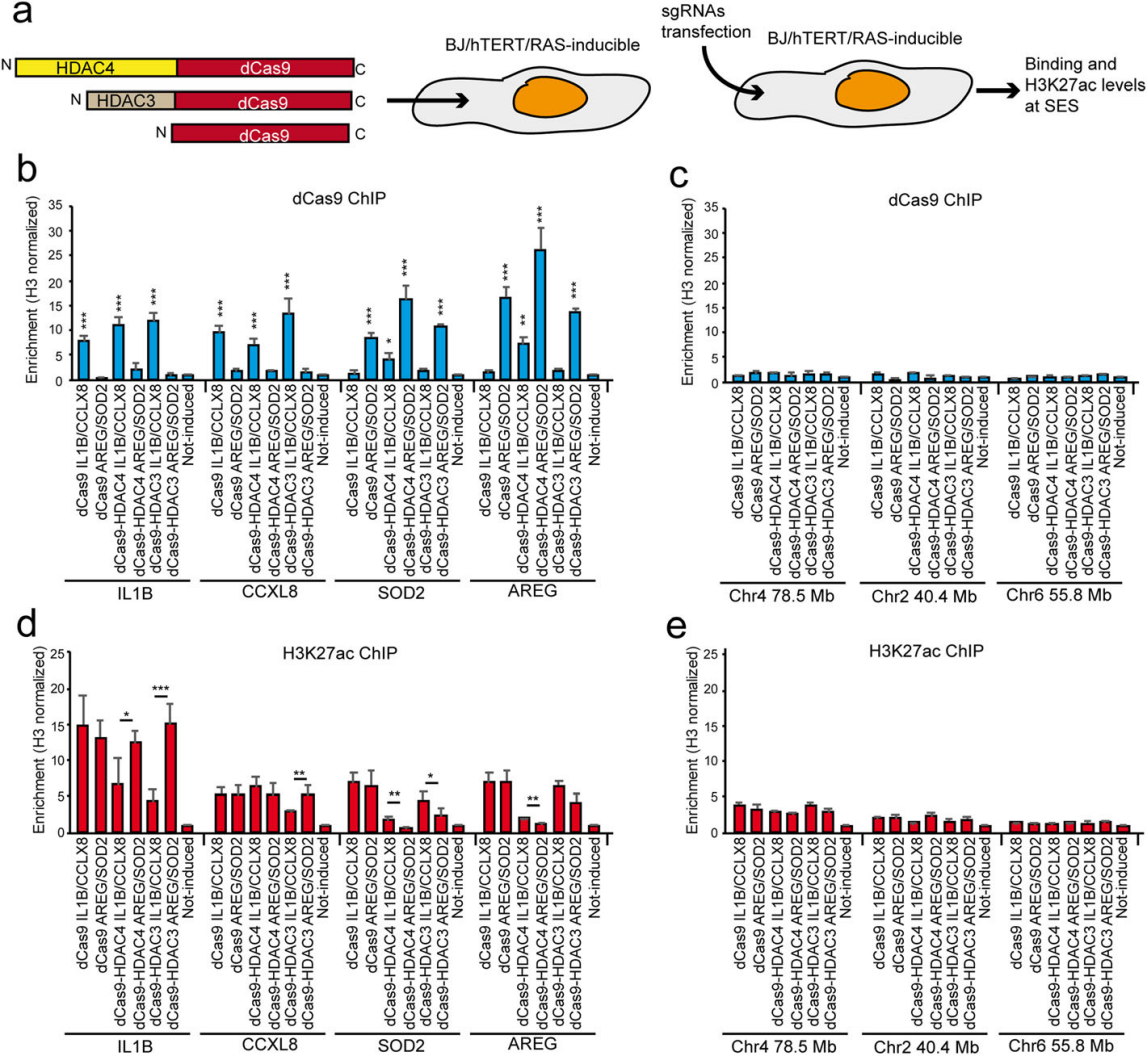
The effect of a forced localization of HDAC3 or HDAC4 on the epigenetic status of selected SES. **a** Schematic representation of the experimental strategy. **b** ChIP-qPCR signals using the anti-Cas9 antibody for the indicated loci in BJ/*hTERT/RAS* cells transfected with the indicated dCas9 chimeras and IVT sgRNAs. The genomic region investigated is indicated with the closest-associated gene. **c** ChIP-qPCR signals using the anti-Cas9 antibody for the indicated loci in BJ/*hTERT/RAS* cells transfected with the indicated dCas9 chimeras and IVT sgRNAs. The genomic region is indicated by the relative coordinates. **d** ChIP-qPCR signals using the anti-H3K27ac antibody for the indicated loci in BJ/*hTERT/RAS* cells transfected with the indicated dCas9 chimeras and IVT sgRNAs. The genomic region investigated is indicated with the closest-associated gene. **e** ChIP-qPCR signals using the anti-H3K27ac antibody for the indicated loci in BJ/*hTERT/RAS* cells transfected with the indicated dCas9 chimeras and IVT sgRNAs. The genomic region is indicated by the relative coordinates. The sgRNAs used are indicated for each bar (e.g., dCas9 IL1B/CXCL8 means that 4sgRNA for IL1B and 4 for CXCL8 were used). Data are reported as mean ± SD and normalized on total H3 signal

HDAC4 and HDAC3 do not bind directly the DNA and are recruited at specific genomic loci by interacting with TFs as part of multiprotein complexes [45]. To further understand the impact of these HDACs on H3K27ac dynamics, we forced their localization to selected SES, independently from their ability to bind these regions in vivo. To this purpose, we delivered HDAC4 and HDAC3 fused to the dead mutant of Cas9 (dCas9 or Cas9^D10A,H840A^) in BJ/*hTERT* cells expressing for 7 days *RAS*, together with 4 sgRNAs to re-direct dCas9-fused proteins on the selected loci (Fig. 7a). We chose *IL1B* and *CCLX8*, as two SES not recognized by HDAC4, as well as *AREG* and *SOD2*, as examples of HDAC4-bound SES. ChIP experiments were performed 48 h later to evaluate both the binding of the different chimeras at the designed loci and H3K27ac levels. The Cas9 ChIP shows that the transfected sgRNAs are able to localize the different chimeras at the expected loci. Importantly, HDAC4 can partially, but substantially, re-direct the Cas9-chimera from *IL1B/CCLX8* to *SOD2/AREG* super-enhancers. This result indicates a strong and autonomous affinity of HDAC4 for these genomic regions (Fig. 7b). To exclude unspecific binding to DNA, we performed the same ChIP on distal genomic regions on the same chromosome. In this case, enrichments were not observed (Fig. 7c), thus confirming the specificity of the targeting. Next, we evaluated H3K27ac levels on the targeted regions. HDAC4-dCas9 chimera reduced the acetylation at *AREG* and *SOD2* SES, whereas it was unable to act on *CCLX8* SES and only partially able to influence *IL1B* SES, even though successfully targeted on these regions (Fig. 7c). By contrast, HDAC3-dCas9 is able to buffer H3K27ac at *CXCL8* and *IL1B* super-enhancers, while it poorly deacetylates H3K27 on the *AREG* and *SOD2* loci. In conclusion, these experiments show that HDAC4 has a strong affinity for the binding and the deacetylation of two HDAC4-specific SES (*SOD2* and *AREG* associated SEs) that we have identified in this manuscript. The forced Cas9-dependent localization of HDAC4 on *IL1B* and *CXCL8*-associated SES leads to some deacetylation only in the case of *IL1B*. This means that the local chromatin environment at certain SES, like *CXCL8*, can impair not only the loading but also the activity of HDAC4 repressive complexes. On the opposite, the HDAC activity of HDAC3-dCas9 is relevant on *IL1B* and *CXCL8* SES, but it is marginal on the tested HDAC4-specific SES (*SOD2*, *AREG*), meaning that HDAC4 assists the activity of HDAC3 not only by directing its binding, but also by acting as recruiting factor for its local action on chromatin.

## Discussion

Enhancer dynamics characterize the appearance of senescence [4, 5]. These extended regions marked by H3K27ac, H3K4me1, and by clusters of transcription factor binding sites supervise the genetic program of senescent cells and particularly the SASP response during OIS [5]. HDAC4 is involved, directly or indirectly, in controlling the H3K27ac levels in approximately 25% of previously defined SES. Moreover, in 9% of them, the direct binding of HDAC4 was observed. Although the list of SES was obtained from studies performed on other cell lines and lineage specific differences exist [14], these data are consistent with an important role of HDAC4 in buffering the full-activation of TEs and SEs involved in senescence. In this scenario, it is important to note the dynamic nature of class IIa HDACs binding to chromatin [14]. A fast turn-over of HDAC4 on chromatin could be an advantage to quickly induce the senescence response. Although dynamically involved in regulating different genomic regions, we have observed that the abrogation of HDAC4 acts rather as an epigenetic priming of senescence that only indirectly and as a later response gives rise to SASP. In fact, even though we forced the loading of HDAC4 onto *CXCL8* and *IL1B* SES, the H3K27ac status was unperturbed or minimally perturbed *(IL1B*) by HDAC4, especially if the repressive effect is compared to its “natural” SES (*AREG* and *SOD2*). Therefore, some SES and TES could be indirectly controlled by HDAC4 in a hierarchical manner. HDAC4 and class IIa HDACs in general can form complexes with class I HDACs and with HDAC3 in particular [18]. We have observed that H3K27ac level at some SE can be modulated by both HDAC4 and HDAC3 whereas, at AREG SE, H3K27ac is modulated only by HDAC4. These preliminary observations deserve further investigations to precisely map the genomic regions bound by these two epigenetic regulators.

Among the SES bound by HDAC4, 66% are enriched for the AP-1 binding motif. AP-1 groups different proteins that can homo- and heterodimerize to constitute the active TF. This complexity guarantees numerous combinations of this TF, which show different transcriptional outputs [46]. It is currently unknown which heterodimers bind and control SES. Nevertheless, our data figured out that HDAC4, assisted by HDAC3, supervises the chromatin status at AP-1 regulated SES, possibly by locally antagonizing p300 activities. In this context, AP-1 has been recently discovered to play relevant roles in pioneering and organizing SES [4, 9]. During senescence entrance, the buffering activity of HDAC4 is lost, as a consequence of its controlled UPS-mediated and GSK3β-assisted degradation. In this manner, TE and SE can orchestrate the transcriptional re-programming. Certainly, the mechanisms engaged by HDAC4 to silence these regions deserve further characterizations and there are several possibilities. HDAC4 could, directly or indirectly, repress AP-1 mediated transcription or, through MEF2, it could also control JUN levels [34] (Additional file 1: Fig. S6f).

The critical role of HDAC4 in senescence is sustained by additional evidence. First, its depletion forces senescence entrance in transformed and pre-transformed cells of different lineages, such as melanomas and leiomyosarcomas. Second, the reintroduction of HDAC4 in a *RAS*-dependent model of OIS counteracts senescence more efficiently with respect to the inhibition of TP53.

Finally, although studies have identified HDAC4 as an epigenetic controller of senescence during OIS and RS, this discovery requires validation, particularly in those senescence responses that do not appear to be characterized by genomic instability, such as NOTCH-induced senescence [47] and embryonic senescence [48].

## Conclusions

Acquiring knowledge on the epigenetic regulation of senescence is fundamental to develop therapeutic approaches to target several age-related diseases including cancer. In summary, our screening aimed to depict class IIa HDACs regulation in senescence has unveiled a complex circuit of events that orbit around HDAC4. Whether the control operated by HDAC4 on the distal regulatory elements such as TE and SE is the sole action to counteracts OIS deserves further studies.

## Methods

### Cell culture and reagents

BJ/hTERT and IMR90 cells, SK-LMS-1 (*TP53*^wt/G245S^*)* leiomyosarcoma cells (previously characterized in [15]), HEK293T, LinXE, Ampho Phoenix, and MEF *HDAC4*^*loxp/loxp*^ cells were cultured as previously described [15] in 10% FBS DMEM (Euroclone). A375 *(TP53*^*wt/wt*^*)* and WM115 [49] (*TP53*^*wt/wt*^) melanoma cells were grown in RPMI. For the conditioning of BJ/hTERT cells, the medium obtained from BJ/*hTERT/HRAS*^*G12V*^ or *HYGRO* cells cultured in 60 mm plates for 8 days was filtered and diluted 1:1 with fresh medium and used to treat cells twice for 96 h, with a change after 48 h. For the experiments performed in hypoxia, cells were grown in hypoxic chambers at 37 °C, 5% CO_2_, and 2% O_2_ (Baker Ruskinn). The following chemicals were used: 250 nM 4-OHT (Sigma-Aldrich), 1 μM Doxycycline (Sigma-Aldrich), 1 μM MG132 (Sigma-Aldrich), 10 μM Chloroquine (Sigma-Aldrich), 50 ng/ml Leptomycin B (Sigma-Aldrich), 0.4% Trypan Blue (Sigma-Aldrich), 200 μM H_2_O_2_ (Sigma-Aldrich), 1 μM PD0332991 (Sigma-Aldrich), 100 nM ABT-263 (Clinisciences), 500 nM JQ1 (Vinci Biochem), and 3 μM A-485 (Vinci Biochem). For luciferase assay, SK-LMS-1 cells HDAC4 wt or KO re-expressing HDAC4 mutants or HDAC3 were transfected with 300 ng pGL3–3XAp1-luc plasmid (Addgene # 40342) and 30 ng pTK-Renilla Luc plasmid. After 48 h, cells were harvest and the assay was performed accordingly to Dual-Glo Luciferase Assay System (Promega). Luminescence was recorder by using Modulus Luminometer Microplate reader (Turner BioSystems) and Luciferase signal was normalized to Renilla signal.

### Generation and culture of *Hdac4*^*fl/fl*^ and *Hdac4*^*−/−*^ murine embryonic fibroblasts

*Hdac4*^*fl/fl*^ mice were previously described [50]. MEFs were generated following standard procedures [51] from 13.5 days-old embryos. Single cell suspensions were expanded in DMEM/10% FBS in hypoxia. 5 × 10^6^ cells were retrovirally infected to express Cre-ER. Not infected cells were removed from culture by puromycin (2 μg/ml, Sigma-Aldrich) selection. The recombination was achieved through the treatment for 48 h with 4-OHT (Sigma-Aldrich). At the end of this incubation, half of the culture was kept in normoxia and half in hypoxia. At each splitting, the total number of cells was counted (Countess II, LifeTechnologies), and the doubling time (dt) was calculated as previously described [52].

### Plasmid construction, transfection, retroviral and lentiviral infection, and silencing

pLENTI-*CRISPR/V2* (Plasmid #52961), p*SpCas9*(BB)-2A-*GFP* (PX458) (Plasmid #48138), p*SpCas9*(BB)-2A-*Puro* (PX459) (Plasmid #62988), pCW-*Cas9* (Plasmid #50661), pCW57/Hygro-MCS1-2A-MCS2 (Plasmid #80922), pBabe-*Puro-IKBalpha* (NFKBIA) S32A/S36A (Plasmid #15291), MSCV-*CreERT2-Puro* (Plasmid #22776), and dCas9-hHDAC3 (Plasmid #98591) were obtained from Addgene. pWZL-*Hygro-HDAC4*^*PAM*^, pWZL-*Hygro-HDAC4*^*PAM*^*-ER*, and pCW-*Hygro-HDAC4*^*PAM*^ were obtained by sub-cloning a mutagenized HDAC4 (QuikChange Site-Directed Mutagenesis Kit, Agilent) into the linearized empty backbones by a restriction-based approach. pWZL-Hygro-ER acceptor plasmid was previously described [53]. *Apple-53BP1trunc* and *H2B-GFP* were sub-cloned respectively in pBABE-*Zeo* and pWZL-*Neo*, NFKBIA-S32A/S36A into pWZL-*Neo-GFP*. pWZL-*Neo-MCS1-2A-MCS2* was obtained by sub-cloning the MCS of pCW57 *Hygro MCS1-2A-MCS2* into pWZL-*Neo* through a recombination-based approach. The generated plasmid was used as acceptor vector for the cloning of *HRAS*^*G12V*^ (*Nhe*I/*Sal*I) and *E1A*/1-143 (*Mlu*I/*Bgl*II-*Bam*HI) to generate pWZL-*Neo-HRAS*^*G12V*^*-*2A*-E1A 1-143*. pWZL-*Hygro-HRAS*^*G12V*^, pBABE-*Puro-HRAS*^*G12V*^, and pBABE-*Puro-myrAKT1* were previously described [32, 54]. pWZL-Neo/HDAC4-GFP, pWZL-*Neo/HDAC4*^Δ600^, pWZL-*Neo/HDAC3*-GFP, pWZL-*Neo/HDAC4*-3xFLAG, pWZL-*Neo/* dCas9 NLS, pWZL-*Neo/HDAC4*-dCas9 NLS, pWZL-*Neo/HDAC3*-dCas9 NLS, pCW-*Puro*/*HRAS*^*G12V*^*,* and *myrAKT1* were obtained by sucloning. All the generated plasmids were checked by restriction and sequencing. The primers used for cloning are listed in additional file 4 Table S3. HDAC4^PAM^ (HDAC4 CDS NM_006037) bears point mutations (V31L) in correspondence to the PAM to prevent its targeting by Cas9. Its efficacy similar to the WT form has been tested on the repression of the target *KLF2* and on the formation of the complex with MEF2D [34].

Transfections, viral infections, and siRNA delivery were done as previously described [17, 54]. The following siRNAs (148 pmol) were used: HDAC4 (CCACCGGAAUCUGAACCACUGCAUU, Invitrogen Stealth) and scramble control siRNA (UAAGGCUAUGAAGAGAUA, Invitrogen Stealth).

### CRISPR*/*Cas9 genome editing

SpCas9 was stably transduced to generate SK-LMS1 *HDAC4*^*−/−*^ clones (76,1231,205,1254,66), and BJ/E1A-RAS *HDAC4*^*−/−*^. SpCas9 was transiently transfected (Lipofectamine 2000, LifeTechnologies) to generate SK-LMS-1 *HDAC4*^*−/−*^ clones (635, 273, 274, 275, 277, 279), A375 *HDAC4*^*−/−*^ clones (304, 401, 150, 1090), and *HDAC4*^*+/−*^ clones (317, 159). The Cas9-resistant HDAC4 (HDAC4^PAM^) was stably expressed prior to the KO (in SK-LMS-1 clones 273,274,275, 277, 279) or continuously re-expressed in a 4-OHT-dependent (SK-LMS-1 clone 66) or DOX-dependent (A375 clones 304, 401, 150, 1090) manner, similarly to what previously described [17]. The sgRNA used are listed in additional file 4 Table S3. Monoclonal cultures were generated by seeding *n* = 1 (SK-LMS-1 and A375) and *n* = 3 (BJ/*E1A-RAS*) cells in each well of 96-well plates (Sarstedt). The successful generation of KO clones was screened by immunoblotting and confirmed by Sanger sequencing.

### Design and delivery of sgRNAs and dCas9 chimeras

Four sgRNAs for the targeting of each SEs were in vitro transcribed (IVT) according to GeneArt™ Precision gRNA Synthesis Kit (Invitrogen). IVT products were treated prior to purification with 5 U of DNAseI (Fermentas) and 10 U of CIP (New England Biolabs), respectively to destroy DNA templates and blunt the dsRNAs inflammatory response [55]. 15 × 10^6^ BJ/hTERT cells expressing RAS were lipofected (Lipofectamine 3000, Thermo Fisher Scientific) with 8 μg of plasmidic DNA encoding Cas9-fused proteins and 8 μg of IVT-treated sgRNAs (2 μg for each sgRNA). Two days later, chromatin was crosslinked, extracted, quantified, and processed for ChIP. The sequence of the sgRNAs used is provided in additional file 4 Table S3.

### Immunofluorescence and immunoblotting

Cells were fixed with 3% paraformaldehyde and permeabilized with 0.3% Triton X-100. The secondary antibodies were Alexa Fluor 488-, 546-, or 633-conjugated anti-mouse and anti-rabbit secondary antibodies (Molecular Probes). Actin was labeled with phalloidin-AF546 or AF-660 (Molecular Probes). For S phase analysis, cells were grown for 3 h with 50 μM Bromodeoxyuridine (BrdU). After fixation, coverslips were treated with HCl (1% and 2%), quenched with Borate, and processed for immunofluorescence. Cells were imaged with a confocal microscope Leica AOBS SP8 or with Leica AF6000 LX. Nuclei were stained with Hoechst 33258.

Cell lysates after SDS-PAGE and immunoblotting on nitrocellulose (Whatman) were incubated with primary antibodies. HPR-conjugated secondary antibodies were obtained from Cell Signaling, and blots were developed with Super Signal West Dura (Thermo Fisher Scientific). Primary and secondary antibodies were removed by using Restore PLUS Western Blot Stripping Buffer (Thermo Fisher Scientific), according to the manufacturer. Unless otherwise indicated, all the immunoblot figures were representative of at least two biological replicates. The primary and secondary antibodies used in this work are listed in the additional file 7, Table S6.

### Proteomics and transcriptomics from in vivo murine aging models

C57BL/6 J female mice were obtained from Shared Ageing Research Models (ShARM, UK). Tissues explanted from 4 months (128 days) and 26 months (774 days) old mice were snap-frozen in liquid nitrogen. For protein lysates generation, subsections of the liver and of the skin were grinded into a powder with a pestle and lysed for 1 h at 4 °C respectively with 400 and 200 μL RIPA lysis buffer for 10 mg of tissue. 4x Laemmli sample buffer was added to the clarified lysates and after boiling the samples were loaded on SDS/PAGE gels. For RNA extraction, 1 ml Tri Reagent (Molecular Research Center) was added to 10 mg of smashed tissues. After 1 h incubation at 4 °C, RNA was recovered by phenol-chloroform extraction/ethanol precipitation and resuspended in 20 μL RNase-free water.

### Immunoprecipitation

Cells were lysed for 10′ into hypotonic lysis buffer (20 mM Tris-HCl pH 7.4, 10 mM KCl, 10 mM MgCl_2_, 1% Triton X-100, 10% glycerol, 50 mM Iac, 1 mM phenylmethylsulfonyl fluoride, 5 mM NaF, 1 mM Na_3_VO_4_), supplemented with protease inhibitors and 10 μM MG-132 and 10 μM G5. Lysates were incubated for 5 h with 1 μg anti-HDAC4 [56] or rabbit IgG and for 1 h with 30 μL slurry protein A (GE). After 4 washes, the immunocomplexes were reversed with 2x Laemmli sample buffer, boiled, resolved by SDS-PAGE, and subjected to western-blotting. One of 100 of total lysate has been collected as input.

### SA-β-gal assay

Cells seeded on coverslips in 12-well plates were fixed for 5′ (PBS 2% formaldehyde/0.2% glutaraldehyde), washed twice with 0.9% NaCl, and stained for 16 h at 37 °C with staining solution: 40 mM citric acid/Na phosphate buffer, 5 mM K4[Fe (CN)6]3H2O, 5 mM K3[Fe (CN)6], 150 mM sodium chloride, 2 mM magnesium chloride, and 1 mg/mL X-gal (Panreact Applichem). Images were acquired with Leica LD bright field optical microscope.

### Transformation assay

Soft agar assay was performed as previously described [32]. Briefly, a total of 0.8 × 10^5^ cells were seeded in 0.3% top agar/DMEM layer above a 0.6% agar/DMEM basement. Fresh medium was added twice/week. After 15 days of culture, the supernatant was discarded and the MTT [3-(4,5-dimethylthiazol-2-yl)-2,5-diphenyltetrazolium bromide] staining (0.5 mg/ml in PBS) was applied for 2 h. Images were acquired with a Leica DN6000 microscope. Foci were automatically counted with Clono Counter.

### RNA extraction and quantitative qRT-PCR

Cells were lysed using Tri Reagent (Molecular Research Center). 1.0 μg of total RNA was DNAseI treated (NEB #T2010) and retro-transcribed by using 100 units of M-MLV Reverse transcriptase (Life Technologies) in the presence of 1.6 μM oligo (dT) (Sigma-Aldrich) and 4 μM Random hexamers (Euroclone). qRT-PCRs were performed using SYBR green technology (KAPA Biosystems). Data were analyzed by comparative threshold cycle (delta delta Ct) using *HPRT* and *GAPDH* or *ACTB* and *GAPDH* as normalizer. The primers used for qRT-PCR are listed in the additional file 6 Table S5.

### RNA array expression and data analysis

Total RNA was purified with Quick-RNA Miniprep (ZymoResearch), amplified according to the specifications of the Illumina TotalPrep RNA Amplification Kit (Ambion) and hybridized on Illumina whole-genome HumanHT-12 v 4.0 chip (Illumina). Acquisition and data analysis were performed as previously described [15]. Principal component analysis (PCA) was performed by using R function prcomp. Differentially expressed genes (DEGs) were called accordingly to the following criteria: |fold change| > 2 and p adj. < 0.05. GSEA analysis in Fig. 2 was performed as previously described [15]. The transcripts defining the “NFκβ,” “SASP” [57], and “RIS upregulated genes” gene sets are listed in the additional file 8, Table S7. Gene list enrichment was performed by interrogating MSigDB collections (BP,C6,CGP,H,MF) with the transcripts associated to promoters (with 2 kb from TSS), TEs or SEs bound by HDAC4; the obtained enrichments were considered significant for p and FDR < 0.05 and if at least three Gene Sets fall in the same category. For the expression levels and Kaplan-Meier analysis of TCGA Skin Cutaneous Melanoma samples, data were retrieved from CBioPortal [58] and expressed as *z*-score. *Z*-scores > |1.75| were selected as cutoff. For bioinformatics analysis in Fig. 3, the following GEO datasets were analyzed: GSE38410, GSE74324, GSE40349, GSE3189, GSE78138, GSE45276, GSE36640, GSE40349, and GSE132569.

### ChIP, library construction, ChIP-seq, and NGS data analysis

Chromatin was obtained from SK-LMS-1 cells, 36 h after or not HDAC4 removal, and immunoprecipitated with 2 μg of anti-H3K27ac, 3 μg of anti-H3K27me3, 4 μg of anti-GFP, 3 μg of anti-H3, and 4 μg of anti-HDAC4 antibodies or control IgG, as previously described [15]. Three independent biological replicates were pulled according to BLUEPRINT requirements and 5 ng of total DNA were used to prepare ChIP-seq libraries, according to TruSeq ChIP Sample Preparation guide (Illumina). Libraries were sequenced on the Illumina HiSeq 2000 sequencer. A minimum overlap of 27% of peaks between independent replicates was obtained and verified before the pulling. The ShortRead R*/*Bioconductor package was used to evaluate the quality of sequencing reads and Bowtie 2 was used to align them to NCBI *GRCh38* human genome reference. Peak calling was performed against input sequences using HOMER for HDAC4 ChIP (“factor” mode) and MACS2 for H3K27ac and H3K27me3 (“sharp” mode and “broad” mode, respectively); gene annotations were performed as previously described [15]. gplots, biomaRt, and Gviz R*/*Bioconductor packages and the deepTools suite were used to generate peak heatmaps and for the visualization of genomic loci. We used the MAnorm method [59] to compare the ChIP-seq signals in the HDAC4 WT and KO conditions. For H3K27ac, H3K27me3, and H3K4me1 signals, the pre- and post-normalization regression lines were very similar and did not require further normalization. For the qPCR-ChIP experiments, normalization was against total H3. Under the time of the analysis H3 protein levels were not decreased. A decrease in H3 was observed only at much later times from HDAC4 depletion-induced senescence.

The H3K27ac and H3K27me3 enriched genomic regions between HDAC4 KO and wt were called according to $\sum \limits_{k=\mathrm{peak}\ \mathrm{summit}}^{\pm 15000}f(k), where\ f(k)=\frac{\mathrm{enrichment}\  KO}{\mathrm{enrichment}\  wt}$. |log_2_(Fc)| ≥ 1 was used as cutoff. As a reference of smooth muscle super-enhancer, the sample_01_066_SE of smooth muscle SE retrieved from the SEdb [60] database (http://www.licpathway.net/sedb/) was used. “SES,” defined according to the ROSE algorithm [61], represents the SEs activated during senescence. SES is the union of the SE identified during OIS [4] and replicative senescence [5] (Additional file 2 Table S1). Closest active algorithm was used for the association between genes and SEs. Liftover tool was used to convert genome coordinates between assemblies and to remap homologous sequences between genomes. The bedtools toolset (--intersect option) [62] was used to identify overlaps of at least one nucleotide between H3K27ac and HDAC4 peaks and the identified SEs and TEs. The investigated classes of genomic elements have been considered as separated, not redundant and not overlapping. The enrichment has been calculated with respect to the genome coverage of each genomic element (length of female haploid human genome: 3184709445 nucleotides). Known and novel motif discovery was performed using the MEME-ChIP tool from the MEME Suite [63]. The following parameters were used: -ccut 0; -order 1; -meme-maxsize 100000000; -meme-minsites 2; -meme-maxsites 100; -mememinw 6; -meme-maxw 10; -meme-nmotifs 10; -meme-mod anr; -dreme-e 0.05; -centrimo-score 5.0; -centrimo-ethresh. Using a 1-order background model to normalize for biased distribution of letters and groups of letters in the analyzed sequences allows to specifically adjust the background for dimer biases (e.g., GC content). The identified enriched motifs were compared to the Jolma2013, JASPAR2018_CORE_vertebrates_non_redundant and uniprobe_mouse databases for annotation. Enrichr (TRRUST) (http://amp.pharm.mssm.edu/Enrichr/) was used for the motif enrichment analysis (Additional file 3, Table S2). For the identification of SE, we used the bedtools toolset to identify overlaps of at least one nucleotide between H3K27ac and H3K4me1 enriched peaks. Afterwards, the ROSE algorithm [61] was used to create stitched enhancers, separating super-enhancers from typical enhancers. Finally, we used the bedtools toolset to identify overlaps of at least one nucleotide between ROSE-defined super-enhancers, HDAC4 enriched peaks, and the list of SES.

### Statistics

For experimental data, Student’s *t* test was employed. The Mann–Whitney test was applied when normality could not be assumed. *p <* 0.05 was chosen as statistical limit of significance. For comparisons between more than two samples, the ANOVA test was applied coupled to Kruskal–Wallis and Dunn’s multiple comparison test. For correlation between two variables, Pearson correlation or Spearman correlation were calculated for normal or non-normal distributions, respectively. Excel and GraphPad Prism were used for routineer analysis, R/Bioconductor packages for large data analysis and heatmap generation. We marked with *p < 0.05, **p < 0.01, and ***p < 0.001. Unless otherwise indicated, all the data in the figures were represented as arithmetic means ± the standard deviations from at least three independent experiments.

## Supplementary Information

**Additional file 1: Supplementary figures**/All supplementary figures.

**Additional file 2: Supplementary Table S1**/bed formatted file of 1053 SES, identified in replicative senescence and OIS.

**Additional file 3: Supplementary Table S2**/List of TFs predicted to regulate the transcripts associated with SEs activated and hyper-acetylated in SK-LMS-1/*HDAC4*^*−/−*^ cells.

**Additional file 4: Supplementary Table S3**/Acetylation ratio calculated between the HDAC4 KO and HDAC4 WT samples, centered on the genomic loci shown in Fig. 4g.

**Additional file 5: Supplementary Table S4**/List of the 382 SES identified by ROSE algorithm in SK-LMS-1 HDAC4 depleted cells.

**Additional file 6: Supplementary Table S5**/List of primers used for cloning, genome editing and PCR.

**Additional file 7: Supplementary Table S6**/List of the antibodies used in this paper.

**Additional file 8: Supplementary Table S7**/List of the genes belonging to the NFκβ, SASP and RIS signatures analyzed in this manuscript.

**Additional file 9.** Review history.

## Data Availability

Raw data corresponding to ChIP-seq experiments are uploaded with GEO accession GSE149644. For reviewers, to access the data, https://www.ncbi.nlm.nih.gov/geo/query/acc.cgi?acc=GSE149644 Enter token clyvsuiwrlqfrgn into the box. Raw data corresponding to DNA microarray experiments are uploaded with GEO accession GSE150427. For reviewers, to access the data, https://www.ncbi.nlm.nih.gov/geo/query/acc.cgi?acc=GSE150427 Enter token kzybqqawhzqhfgd into the box.
