# Supplementary material for: HDAC4 degradation during senescence unleashes an epigenetic program driven by AP-1/p300 at selected enhancers and super-enhancers
Source: Genome Biol. 2021 May 10;22:129. doi: 10.1186/s13059-021-02340-z (PMC8108360; doi:10.1186/s13059-021-02340-z)
Supplement: Supplementary file 1 — Additional file 1: Supplementary figures/All supplementary figures. [file 13059_2021_2340_MOESM1_ESM.pdf]

HDAC4 degradation during senescence unleashes an epigenetic program driven by AP-1/p300 at selected enhancers and super-enhancers

Eros Di Giorgio[§], Harikrishnareddy Paluvai[§], Emiliano Dalla[§], Liliana Ranzino[§], Alessandra Renzini[#], Viviana Moresi[#], Martina Minisini [§], Raffaella Picco[§], Claudio Brancolini[§*]

[§]Department of Medicine, Università degli Studi di Udine, p.le Kolbe 4, 33100 Udine, Italy

[#]DAHFMO Unit of Histology and Medical Embryology, Sapienza University of Rome, via Antonio Scarpa 16, 00161, Rome, Italy

[*]Correspondence and Lead Contact: claudio.brancolini@uniud.it

ADDITIONAL FILE 1 .pdf

Supplementary figures

All supplementary figures

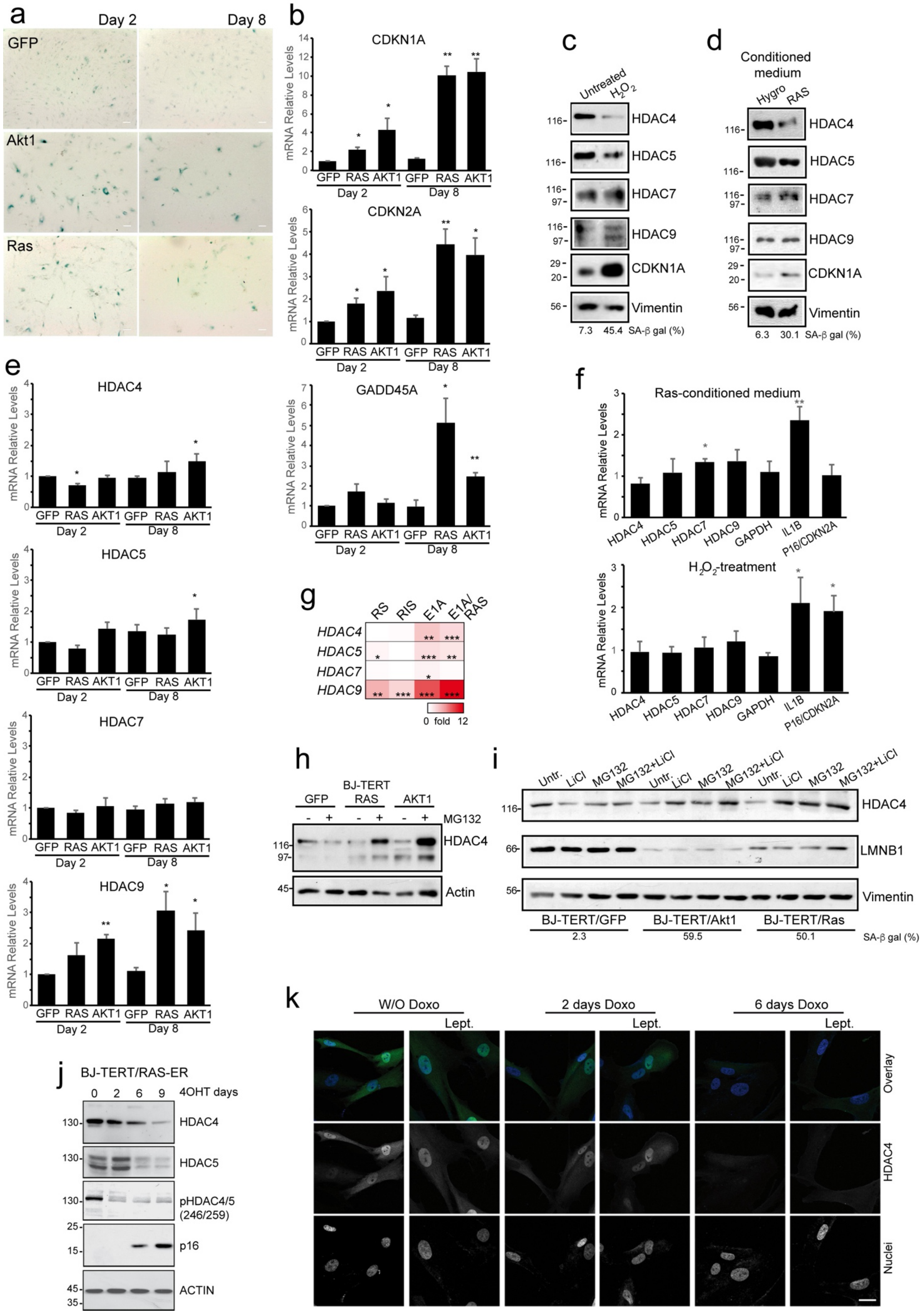
a
Day 2
Day 8
GFP
Akt1
Ras
b
CDKN1A
mRNA Relative Levels
GFP RAS AKT1 GFP RAS AKT1
Day 2
Day 8
CDKN2A
GADD45A
c
Untreated
H2O2
HDAC4
HDAC5
HDAC7
HDAC9
CDKN1A
Vimentin
7.3 45.4 SA-β gal (%)
d
Conditioned medium
Hygro
RAS
6.3 30.1 SA-β gal (%)
e
HDAC4
HDAC5
HDAC7
HDAC9
f
Ras-conditioned medium
H2O2-treatment
HDAC4 HDAC5 HDAC7 HDAC9 GAPDH IL1B P16/CDKN2A
g
RS RIS E1A E1A/RAS
HDAC4
HDAC5
HDAC7
HDAC9
0 fold 12
h
BJ-TERT
GFP RAS AKT1
MG132
HDAC4
Actin
i
Untr. LiCl MG132 MG132+LiCl
HDAC4
LMNB1
Vimentin
BJ-TERT/GFP
BJ-TERT/Akt1
BJ-TERT/Ras
2.3 59.5 50.1 SA-β gal (%)
j
BJ-TERT/RAS-ER
0 2 6 9 4OHT days
HDAC4
HDAC5
pHDAC4/5 (246/259)
p16
ACTIN
k
W/O Doxo
2 days Doxo
6 days Doxo
Lept.
Overlay
HDAC4
Nuclei

Figure S1. Class IIa HDACs are dysregulated in different types of cellular senescence.

a. Microscopic images of SA-β-gal stained BJ/hTERT cells expressing the indicated transgenes for 2 or 8 days. Scale bar 50 µm.

b. mRNA expression levels of the indicated genes in the indicated BJ/*hTERT* cells. Mean ± SD; n = 3.

c. Immunoblot analysis using the indicated antibodies of BJ//*hTERT* cells treated with H_2O_2 (200µM) for 2h and harvested after 4 days. SA-β-gal is indicated. Vimentin was used as loading control.

d. Immunoblot analysis using the indicated antibodies of BJ/*hTERT* grown for 48 h. with a conditioned medium (1:1 conditioned/fresh medium) 48h) obtained from BJ/hTERT cells stably expressing $HYGRO^R$ or $HRAS^{G12V}$. SA-β-gal is indicated. Vimentin was used as loading control.

e. mRNA expression levels of the indicated genes in BJ/hTERT cells generated and treated as described in figure S1a. Mean ± SD; n = 3.

f. mRNA expression levels of the indicated genes in BJ/hTERT cells generated and treated as described in figures S1c/d. Mean ± SD; n = 3.

g. Heatmap summarizing the mRNA expression levels of Class IIa HDACs in respect to control cells. Mean ± SD; n = 3. RS= replicative senescence; RIS= RAS-induced senescence.

h. Immunoblot analysis of HDAC4 and Actin in the input (1/100 total lysate) related to the immunoprecipitation shown in figure 1i.

i. Immunoblot analysis of HDAC4 and LMNB1 in BJ/*hTERT* cells expressing for 6 days the indicated transgenes and treated for the last 12h with LiCl (10mM) and/or MG132 (1µM), as indicated. Vimentin was used as loading control.

j. Immunoblot analysis using the indicated antibodies of BJ//*hTERT-RAS-ER* cells treated with 4-OHT for the indicated times. The indicated antibodies were used to evaluate the appearance of senescence and the phosphorylation of class IIa HDACs.

k. Immunofluorescence analysis of HDAC4 localization in BJ//*hTERT-RAS-ER* cells treated with 4-OHT for the indicated times. Confocal images were obtained with a Leica LSM SP8 and are shown in pseudocolors. Bar 25µm.

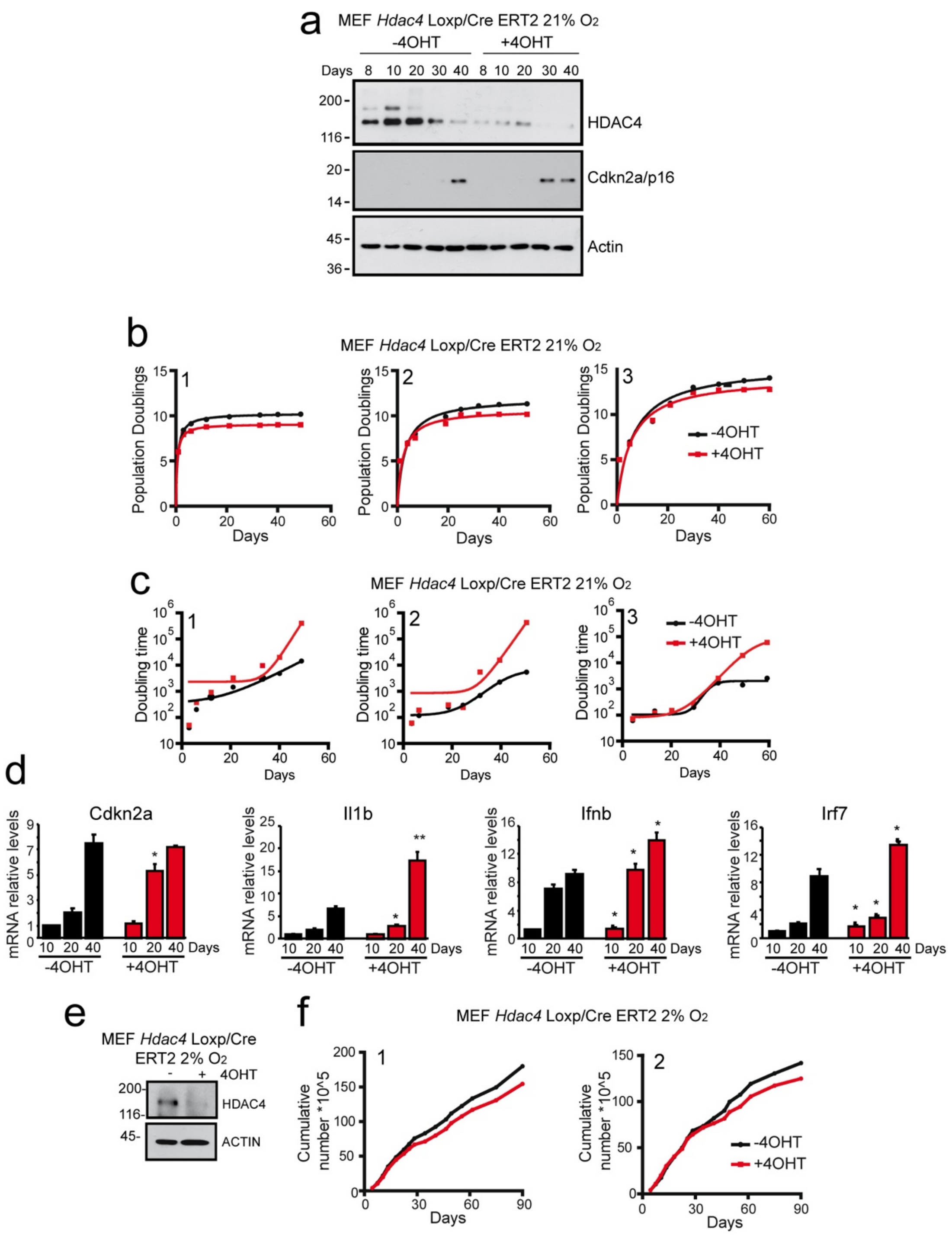

Figure S2. The forced knock-out of HDAC4 allows premature senescence entrance.

a. Immunoblot analysis of HDAC4 and p16 levels in MEF$^{loxp/loxp}$ x CreER T2 cells, maintained in normoxia and treated or not for 48h with 0.25µM 4-OHT at day 6 of culture and harvested after regular splitting at the indicated days. Actin was used as loading control.

b. Analysis of the cumulative population doublings **(E)** or the doubling time **(F)** observed during the culture of thee independent batches of MEF$^{loxp/loxp}$ x CreER T2 cells maintained as described in figure S2a. Third order polynomial regression curves are shown.

c. Analysis of the doubling time observed during the culture of thee independent batches of MEF$^{loxp/loxp}$ x CreER T2 cells maintained as described in figure S2a. Sigmoidal regression curves are shown.

d. mRNA expression levels of the same MEF cells described in figure S2a. Mean ± SD; n = 2. For significance calculation, a paired comparison between WT and *Hdac4*$^{-/-}$ cells was made for each growth point.

e. Immunoblot analysis of HDAC4 levels in MEF$^{loxp/loxp}$ x CreER T2 cells, maintained in hypoxia and treated or not for 48h with 0.25µM 4-OHT at day 6 of culture and harvested after regular splitting at day 30. Actin was used as loading control.

f. Analysis of the cumulative population doublings observed during the culture of two independent batches of MEF$^{loxp/loxp}$ x CreER cells maintained as described in figure S2e.

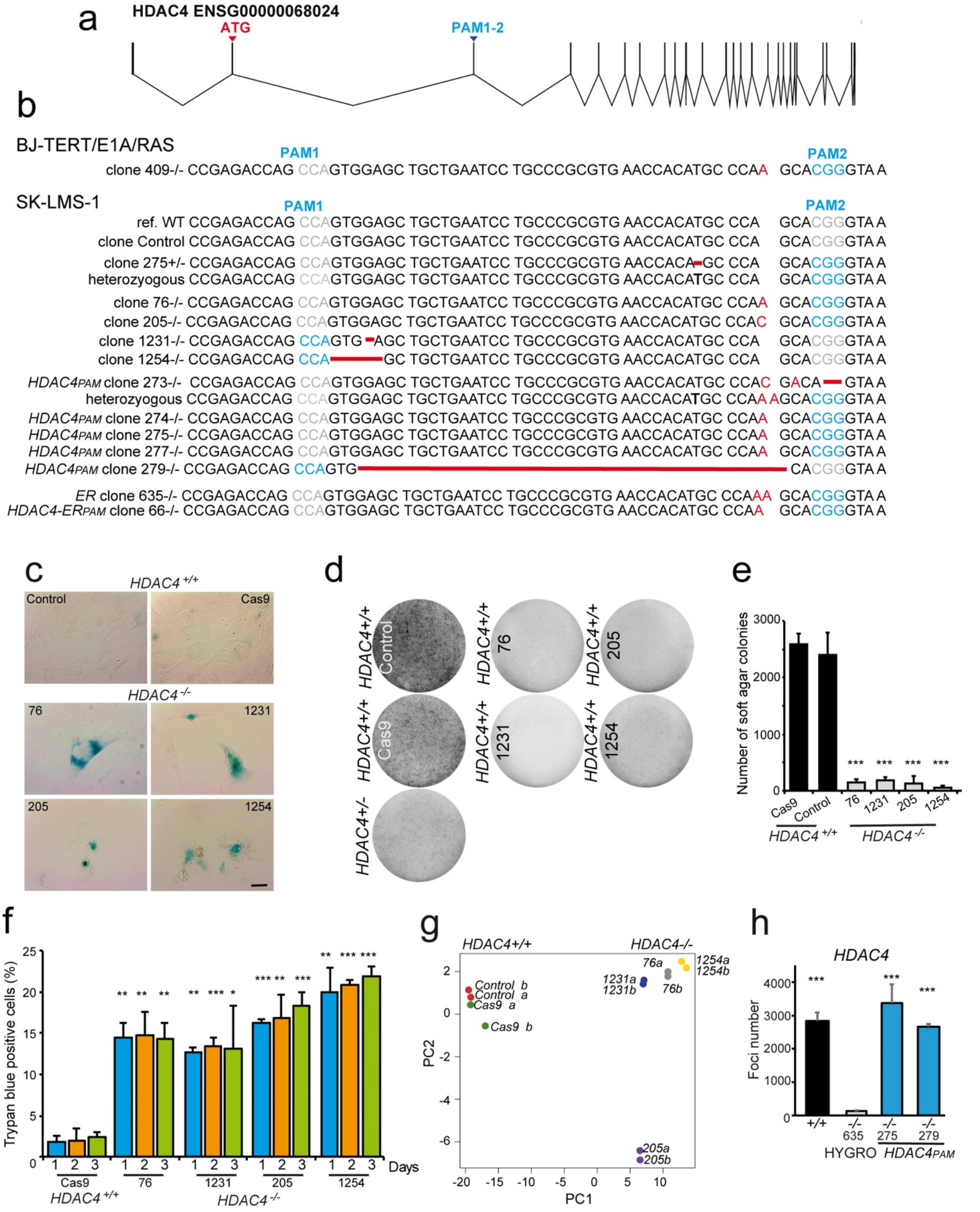

Figure S3. HDAC4 depletion causes a senescence-like cell cycle arrest.

a. Schematic representation of HDAC4 genomic organization with indicated: the exons (vertical bars), the introns (junctions between the bars) and the PAM sequences utilized for the CRISPR/Cas9 genome editing.

b. Genomic sequences of the *HDAC4*$^{-/-}$ BJ/RAS/E1A and SK-LMS-1 cells used in this study.

c. Microscopic images of SA-β-gal stained SK-LMS-1 clones, as indicated.

d. Representative images of MTT stained foci formed from the indicated SK-LMS-1 clones and grown for 15 days in soft-agar.

e. Quantification of the stained foci displayed in Fig. S3e. Data are presented as mean ± SD; n = 4.

f. Histogram representing the percentage of TB positivity in the indicated SK-LMS-1 clones. Mean ± SD; n = 3.

g. PCA analysis performed on the expression profile of the indicated SK-LMS-1 clones.

h. Number of foci generated in soft-agar Analysis by WT or *HDAC4*$^{-/-}$ SK-LMS-1 cells expressing *HYGRO*^{R} (clone 635) or *HDAC4* (clones 275 and 279). Mean ± SD; n = 4. The significance in relative to the KO clone 635.

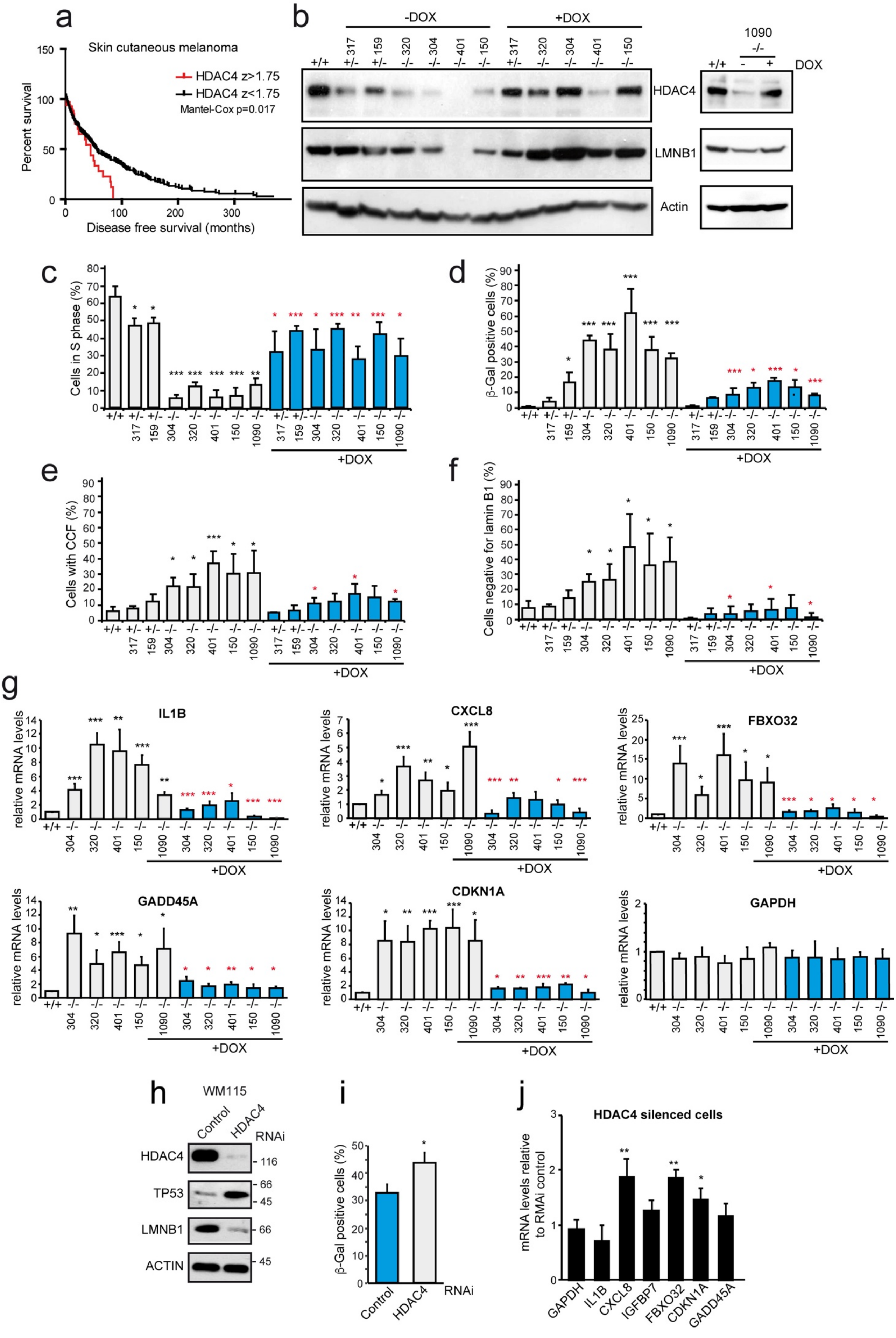

a
Skin cutaneous melanoma
HDAC4 z>1.75
HDAC4 z<1.75
Mantel-Cox p=0.017
Percent survival
Disease free survival (months)
b
-DOX
+DOX
HDAC4
LMNB1
Actin
1090
DOX
c
Cells in S phase (%)
+DOX
d
β-Gal positive cells (%)
+DOX
e
Cells with CCF (%)
+DOX
f
Cells negative for lamin B1 (%)
+DOX
g
IL1B
CXCL8
FBXO32
GADD45A
CDKN1A
GAPDH
relative mRNA levels
+DOX
h
WM115
Control
HDAC4
RNAi
HDAC4
TP53
LMNB1
ACTIN
i
β-Gal positive cells (%)
Control
HDAC4
RNAi
j
HDAC4 silenced cells
mRNA levels relative to RMAi control
GAPDH
IL1B
CXCL8
IGFBP7
FBXO32
CDKN1A
GADD45A

Figure S4. Characterization of the role played by HDAC4 in sustaining senescence escape and melanomagenesis.

a. Kaplan-Meier survival analysis related to the expression levels of HDAC4 (z-score>1.75, n=25) in 425 TCGA melanoma samples. Median months overall survival: 47.54 and 81.20 respectively in HDAC4 high and HDAC4 low clusters of patients.

b. Immunoblot analysis of HDAC4 and LMNB1 in the indicated A375 clones, re-expressing or not a doxycycline (DOX)-inducible CRISPR/Cas9 resistant form of HDAC4 ($HDAC4^{PAM}$). Actin was used as loading control.

c. Analysis of the percentage of BrdU positive cells. The significance is relative to WT cells (black *) or to clones grown in absence of DOX (red *). Mean ± SD; n=4.

d. Analysis of the percentage of SA-β-gal positive cells. The significance is relative to WT cells (black *) or to clones grown in absence of DOX (red *). Mean ± SD; n=4.

e. Analysis of the percentage of cells displaying chromatin cytoplasmic fragments (CCFs). The significance is relative to WT cells (black *) or to clones grown in absence of DOX (red *). Mean ± SD; n=4.

f. Analysis of the percentage of cells with down-regulated/absent LMNB1. The significance is relative to WT cells (black *) or to clones grown in absence of DOX (red *). Mean ± SD; n=4.

g. mRNA expression levels of the indicated genes in the indicated A375 clones, grown for 4days in the presence or absence of DOX. The significance and cell treatments are as explained in Fig. S4F. Mean ± SD; n=4.

h. Immunoblot analysis in WM115 cells silenced or not for HDAC4 (72h), using the indicated antibodies. Actin was used as loading control.

i. Analysis of the percentage of SA-β-gal positivite WM115 cells, after the silencing of HDAC4 for 72 hours. Mean ± SD; n=3.

j. mRNA expression levels of the indicated genes in WM115 silenced for HDAC4, in respect to siRNA Control. Mean ± SD; n=3.

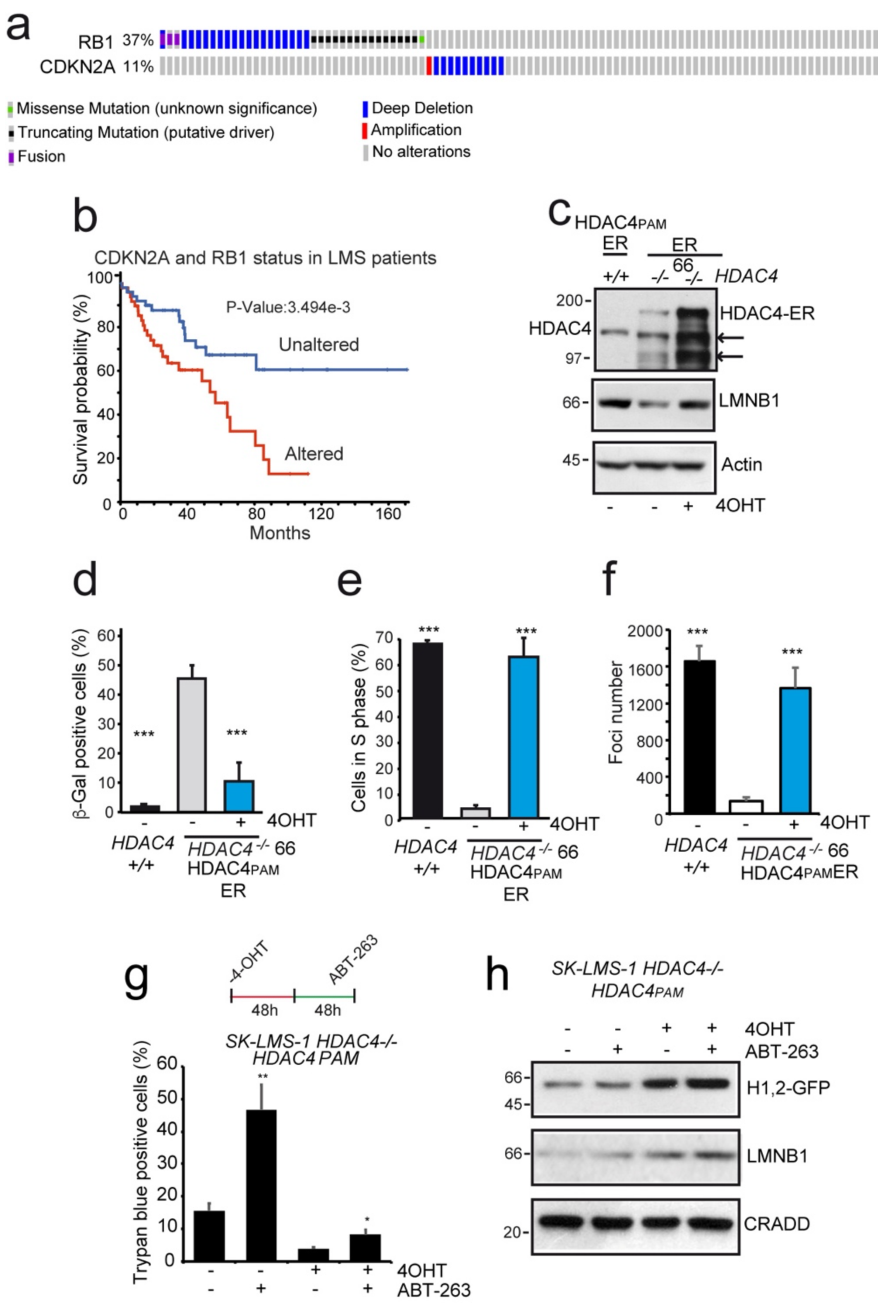

Figure S5. Characterization of the pro-senescent properties arising from HDAC4 depletion in the established inducible rescue models.

a. Oncoprint of the mutational status of *RB1* and *CDKN2A* in 100 LMS TCGA samples.

b. Kaplan-Meier survival analysis related to the mutational status of RB1 and CDKN2A (altered group n=48, unaltered n=52; median months overall survival: 56.61 and >170 respectively in altered and unaltered clusters of patients.

c. Immunoblot analysis in SK-LMS-1 WT or *HDAC4*$^{-/-}$ (clone 66) cells re-expressing a tamoxifen inducible *HDAC4*^{PAM}*-ER*. Arrowheads point to HDAC4 cleavage products observed in HDAC4-ER expressing cells. Actin was the loading control.

d. Analysis of SA-β-gal positive SK-LMS-1 cells *HDAC4*$^{+/+}$, *HDAC4*$^{-/-}$ or *HDAC4*$^{-/-}$ re-expressing the tamoxifen inducible *HDAC4*^{PAM}*-ER*. Mean ± SD; n=4.

e. Analysis of BrdU positive SK-LMS-1 cells *HDAC4*$^{+/+}$, *HDAC4*$^{-/-}$ or *HDAC4*$^{-/-}$ re-expressing the tamoxifen inducible *HDAC4*^{PAM}*-ER*. Mean ± SD; n=4.

f. Analysis of foci formation after growing in soft agar SK-LMS-1 cells *HDAC4*$^{+/+}$, *HDAC4*$^{-/-}$ or *HDAC4*$^{-/-}$ re-expressing the tamoxifen inducible *HDAC4*^{PAM}*-ER*. Mean ± SD; n=4.

g. The senolytic drug ABT-263 was applied to SK-LMS-1 *HDAC4*$^{-/-}$ *HDAC4PAM-ER* cells as indicated to trigger apoptosis. Trypan blue positive cells are shown. Mean ± SD; n = 3.

h. Immunoblot analysis of HDAC4 and H1.2 GFP in the same cells described in Fig. S5h. CRADD was used as loading control.

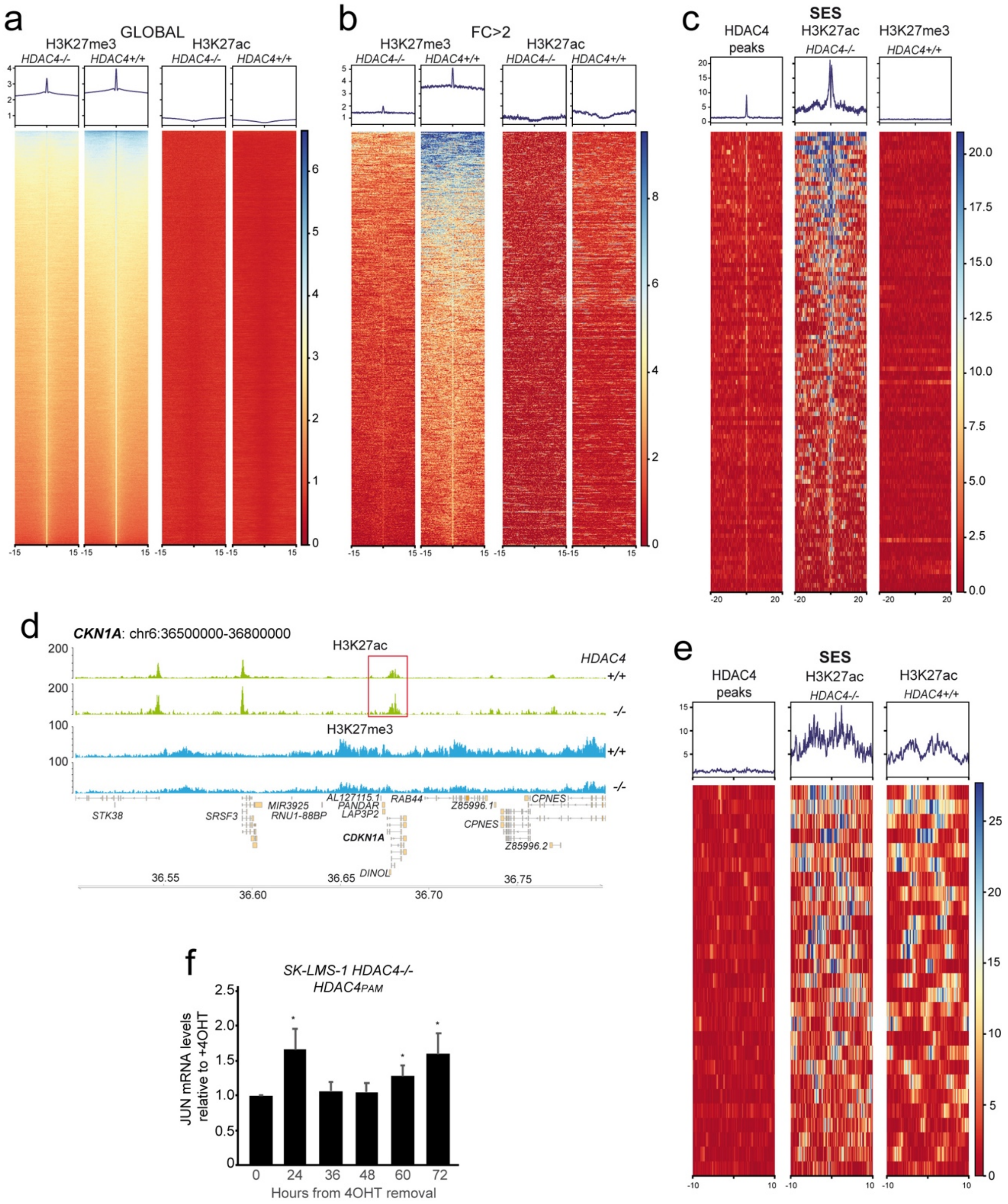

Figure S6. HDAC4 supervises enhancers and super-enhancers activated early during senescence.

a. Heat-map of the 139200 H3K27me3 enriched peaks ("GLOBAL") and of the associated H3K27ac signal in the same regions in the indicated SK-LMS-1 cells. The analysis was performed in a region of ±15Kb around peak summit.

b. Heat-map of the 1205 H3K27me3 peaks ("FC>2") displaying an overall signal ratio (WT/KO)>2 and of the associated H3K27ac signal in the same regions. The analysis was performed in a region of ±15Kb around peak summit.

c. Heat-map of the HDAC4 (WT), H3K27ac (KO), and H3K27me3 (WT) signals in the 91 SES containing HDAC4 enriched peaks. The analysis was performed in a region of ±20Kb around peak summit.

d. Detailed view of the H3K27ac and H3K27me3 levels in the *CDKN1A* locus in WT and HDAC4 KO SK-LMS-1 cells.

e. Heat-map of the HDAC4 (WT), H3K27ac (KO), and H3K27ac (WT) signals in SES of SK-LMS-1 cells containing HDAC4 enriched peaks. The analysis was performed in a region of ±10Kb around peak summit. The ROSE algorithm was used to separate super-enhancers from typical enhancers. SE defined in SK-LMS-1/*HDAC4*$^{-/-}$ cells expressing the tamoxifen inducible version of HDAC4 (WT) were subtracted from SE defined in the same cells grown for 36h without 4-OHT. The obtained 363 SE represent the SES of SK-LMS-1 cells. These SES were next selected for the presence of HDAC4 peaks (n=27).

f. JUN mRNA expression levels in SK-LMS-1 *HDAC4*$^{-/-}$ cells, re-expressing (+4-OHT) or not (-4-OHT) HDAC4^{PAM}-ER. The levels are relative to SK-LMS-1 *HDAC4*$^{-/-}$HDAC4^{PAM}-ER grown in the presence of 4OHT. Mean ± SD; n = 3.
