# Supplementary material for: HDAC4 degradation during senescence unleashes an epigenetic program driven by AP-1/p300 at selected enhancers and super-enhancers
Source: Genome Biol. 2021 May 10;22:129. doi: 10.1186/s13059-021-02340-z (PMC8108360; doi:10.1186/s13059-021-02340-z)
Supplement: Supplementary file 9 — Additional file 9. Review history. [file 13059_2021_2340_MOESM9_ESM.docx]

Review History

**First round of review**

**Reviewer 1**

**Are you able to assess all statistics in the manuscript, including the appropriateness of statistical tests used?**

Yes, and I have assessed the statistics in my report.

**Comments to author:**

In this manuscript, Giorgio et al identify HDAC4 as a key regulator of enhancers and superenhancers in senescence. They show that HDAC4 is polyubiquitinated and targeted for proteosomal degradation right before senescence establishment which then allows AP1/p300 to rapidly dominate these sites and establish enhancers and superenhancers. Transformed cells that bypass oncogene-induced senescence can be induced to re-establish senescence by deletion of HDAC4.

The authors have performed a variety of intricate genetic manipulations (CRISPR/Cas9, Cas9-resistant mutants, rescue experiments, ChIP-seq etc.) to show the role of HDAC4 and its potential therapeutic value in several senescence-bypass systems.

The overall conclusions about HDAC4 function in senescence bypass are interesting. But some directed re-analysis is required to support their conclusions. These and some other concerns are listed below:

Major concerns

1.	Why were only female mice used in the western blots in Figure 1C?

2.	In Figure 1h – why wasn’t p62 upregulated in the chloroquine treated and MG132+chloroquine treated cells?

3.	The authors have compiled a list of SES and TES from primary fibroblast cells and a smooth muscle lineage (not senescent). This cross-comparison could be problematic as enhancers are highly cell-type specific. Additionally, enhancers are also marked by H3K4me1. It would be best if the authors generated this ChIP-seq dataset and then called their own enhancers (TES and SES) using both established marks (H3K4me1 and H3K27ac) in senescent cells/upon removal of HDAC4/reintroduction of HDAC4.

4.	Figure 4a/b – how do the authors think the decrease of H3K27me3 globally occurs with HDAC4 depletion? From the figure legend, the H3K27ac and H3K27me3 heatmaps are focused on different sites in the genome. Is HDAC4 recruited to these different sites? It would be best to focus on the same sites and show the opposite directions of H3K27ac and H3K27me3 change. Additionally, it would be fitting if the authors focused on genomic sites where HDAC4 is recruited by ChIP-seq. In other words, can Figure 4d (HDAC4 regions) be shown as a heatmap?

5.	Figure 4g – the reduction in acetylation after induction of HDAC4 looks modest. Do the authors have a quantification (such as area under the curve) of this reduction across the SESs regulated by HDAC4?

6.	What was the background chosen for motif search? It would be appropriate to consider a union of H3K27ac peaks rather than whole genome as ChIP-seq data often has GC biases.

7.	It is not clear if the ChIP signals throughout the manuscript (for example in Figure 4g, 6c) were normalized to H3. This is very important particularly in senescent cells as they are known to downregulate histones.

Minor concerns

1.	Please correct typos throughout manuscript, for example “knowledges”, “differentiative” on page 3, “telomers” on page 4, “regulative” on page 7, “knowledges” on page 13 etc.

2.	In the Background section, I would not really mention “SWI/SNF/p300 complex” as they are not a part of the same complex but rather interact with each other. Additionally, the phrase “senescent genetic program” should be replaced. Do the authors mean senescence gene expression program?

3.	Figure 1d-g would benefit from a discussion of why the authors see different effects in different cells used for OIS. For example, why does HDAC9 go up in BJ-TERT cells but decrease in IMR90 cells?

4.	The authors mention on page 6, “As expected, NFKB inhibition only partially recovers the senescent phenotype in terms of SA-β-gal positivity and progression through S-phase (Fig. 3j/k).” Could the authors provide a reference? It might be useful to clarify the difference between p53/pRb inhibition versus NFkB inhibition and their distinct effects on senescence in the text.

5.	Could the authors clarify what they mean by “Next, ChIP-seq was performed after 36h from HDAC4 depletion” on page 8? Isn’t this ChIP-seq from HDAC4-/- cells?

6.	On page 9 – please revise the sentence “Moreover, 13.5% of HDAC4 peaks were found to directly control the chromatin folding in SES (Fig. 4e)”. This data indicates HDAC4 peaks are found in SES but doesn’t imply anything about folding and may be misleading to readers. Please also revise the sentence on line 208 of page 8.

7.	Please describe the experimental details of AP1 transcriptional activity (Figure 6b) in greater detail in the Methods.

8.	dCas9 ChIP should be normalized to input not H3.

9.	In general it would be helpful to readers to simplify the wording in the text. Sometimes, the sentences are convoluted and difficult to follow.

**Reviewer 2**

**Are you able to assess all statistics in the manuscript, including the appropriateness of statistical tests used?**

Yes, and I have assessed the statistics in my report.

**Comments to author:**

In this study, the authors investigated the role of histone deacetylase 4 (HDAC4) and its paralogs in regulating epigenetic programs during cell senescence and animal aging. The authors discovered that senescence and aging trigger gradual decrease in the expression of HDAC4 and its paralogs, HDAC7 and HDAC9. Then the underlying epigenomic impact was investigated by ChIP-Seq. The results indicate that HDAC4 acts through c-Jun/Fos and affect acetylation and methylation of histone H3 at lysine 27. The role of these enzymes in senescence and aging is totally unexpected, and thus represents a major advance for the study of these enzymes. The data is of high quality and the presentation is very clear as well.

I have a few comments for the authors to consider in revising the manuscript:

1) Phosphorylation is a major regulatory event for these enzymes, so I would like to see some data in this regard.

2) MEF2 is a key DNA-binding partner, whereas c-Jun/Fos is not so well established. On the other hand, MEF2 may regulate the expression c-Jun/Fos, so I would suggest to analyze whether the impact on H3K27 modifications are direct.

3) There are reports that HDAC4 recruits HDAC3 for deacetylation and that HDAC3 is known to deacetylase H3K27, so the authors may need to sort this out as well.

4) For immunoblotting mouse aging (Fig. 1C), I would suggest to add results on muscle and brain as HDAC4 is much more important in those tissues.

5) The title is a bit long and could be shortened to make it more catchy. Also, the word 'anticipate" on page 5 should be replaced with words such as "promote", "stimulate" and "enhance."

**Authors Response**

**Point-by-point responses to the reviewers’ comments:**

In this manuscript, Giorgio et al identify HDAC4 as a key regulator of enhancers and superenhancers in senescence. They show that HDAC4 is polyubiquitinated and targeted for proteosomal degradation right before senescence establishment which then allows AP1/p300 to rapidly dominate these sites and establish enhancers and superenhancers. Transformed cells that bypass oncogene-induced senescence can be induced to re-establish senescence by deletion of HDAC4.

The authors have performed a variety of intricate genetic manipulations (CRISPR/Cas9, Cas9-resistant mutants, rescue experiments, ChIP-seq etc.) to show the role of HDAC4 and its potential therapeutic value in several senescence-bypass systems.

The overall conclusions about HDAC4 function in senescence bypass are interesting. But some directed re-analysis is required to support their conclusions. These and some other concerns are listed below:

Major concerns

1.	Why were only female mice used in the western blots in Figure 1C?

*The question of sexual dimorphism in ageing is interesting. It is well known that the ageing process shows gender effect (Hum Genet. 2020;139:333). In humans, female longevity tends to exceed male longevity, which is why we selected female mice (Psychol Health Med. 2015;20:129). However, these aspects are beyond the scope of our manuscript. Overall, we are aware that the observation in aged mice is preliminary and further studies are needed to better understand the contribution of HDAC4 and of class IIa HDACs to the ageing process in vivo.*

2.	In Figure 1h – why wasn’t p62 upregulated in the chloroquine treated and MG132+chloroquine treated cells?

*We thank the Reviewer for this point. We verified the original immunoblot and noted that the orientation for p62 was inverted. We have re-organized the figure with the right orientation. We apologize for the mistake.*

3.	The authors have compiled a list of SES and TES from primary fibroblast cells and a smooth muscle lineage (not senescent). This cross-comparison could be problematic as enhancers are highly cell-type specific. Additionally, enhancers are also marked by H3K4me1. It would be best if the authors generated this ChIP-seq dataset and then called their own enhancers (TES and SES) using both established marks (H3K4me1 and H3K27ac) in senescent cells/upon removal of HDAC4/reintroduction of HDAC4.

*As requested, we mapped SE in SK-LMS-1 cells by performing H3K4me1 ChIP-seq in cells WT and KO for HDAC4 at 36 hours from HDAC4 depletion. To rank SE, we applied the superenhancers (ROSE) algorithm. We are interested in characterizing the core regulative elements of the senescence response. These elements should be conserved among different cell lines. Hence, we crossed the list of SE modulated after HDAC4 depletion in SK-LMS-1 cells, with the list of SES previously characterized. We obtained a good overlap. 39% of SE regulated by HDAC4 in SK-LMS-1 were previously characterized as SES in other cell lines. We thank the reviewer for this request. The results obtained further strengthen the importance of HDAC4 in controlling SES dynamics.*

*We have inserted the following text in the revised version of the manuscript, modified figure 5a and included a new additional table (Table S4).*

*Old text.*

*Among the 91 SES directly regulated by HDAC4, 63 of them bear the AP-1 consensus in correspondence to HDAC4 binding sites (Fig. 5a/b).*

*New text.*

*H3K27ac peaks modulated by HDAC4 can be found in 256 SES defined in other cell lines. Among them, 91 SES are directly regulated by HDAC4 and 63 of them bear the AP-1 consensus in correspondence to HDAC4 binding sites (Fig. 5a). Since SE can be cell type specific, to strengthen the importance of HDAC4 in the regulation of SES, ChIP-seq experiments were performed to map H3K4me1 distribution in SK-LMS-1 WT cells or at 36h from HDAC4 depletion. Using the ROSE algorithm 980 SE modulated by HDAC4 were identified (Additional file 5: Table S4). 39% of them were previously defined as SES in other cell lineages (Fig. 5a). Relying on the importance of HDAC4 in controlling SES dynamics, we next explored the role of AP-1 in the modulation of SES under HDAC4 influence. We focused the analysis on the 63 SES that bear the AP-1 binding sites and show variations in H3K27 acetylation. One third...*

*We have also inserted in the methods section the new analysis. For the identification of SE, we used the bedtools toolset to identify overlaps of at least one nucleotide between H3K27ac and H3K4me1 enriched peaks. Afterwards, the ROSE algorithm (Warren A. et al Cell 153, 307-319, April 11, 2013) was used to create stitched enhancers, separating super-enhancers from typical enhancers. Finally, we used the bedtools toolset to identify overlaps of at least one nucleotide between ROSE-defined super-enhancers, HDAC4 enriched peaks and the list of SES.*

4. Figure 4a/b – how do the authors think the decrease of H3K27me3 globally occurs with HDAC4 depletion? From the figure legend, the H3K27ac and H3K27me3 heatmaps are focused on different sites in the genome. Is HDAC4 recruited to these different sites? It would be best to focus on the same sites and show the opposite directions of H3K27ac and H3K27me3 change.

*We do not have experimental data explaining how HDAC4 could influence H3K27me3 levels. Probably different mechanisms are involved. In some of these H3K27me3 regions HDAC4 binding is detected and therefore it could be, in some undefined manner, directly implicated. It is also plausible that an indirect mechanism is involved, perhaps related to the induction of senescence. Further studies are required to clarify this point. As the reviewer requested, we have modified figure 4a/b and generated a new additional file with new figure S6a/b). It is evident that variations in H3K27ac and H3K27me3 involve different genomic regions. Accordingly, we have modified the text.*

*Old Text*

*Next, ChIP-seq was performed after 36h from HDAC4 depletion. Genome-wide levels of H3K27ac and H3K27me3 show moderate and focused decreases of H3K27me3 and locus specific increases of H3K27ac after HDAC4 depletion (Fig. 4a/b).*

*New text*

*Next, ChIP-seq was performed after 36h from HDAC4 depletion to monitor H3K27ac and H3K27me3 variations. Genome-wide analysis shows locus specific increases of H3K27ac levels in cells knocked-out for HDAC4. These loci do not show overt variations in H3K27me3 (Fig. 4a/b). In parallel, HDAC4 depletion triggers moderate decreases of H3K27me3 levels in loci that do not show overt variations in H3K27 acetylation (Additional file 1: Fig. S6a/b).*

Additionally, it would be fitting if the authors focused on genomic sites where HDAC4 is recruited by ChIP-seq. In other words, can Figure 4d (HDAC4 regions) be shown as a heatmap?

*We have performed the analysis that is included in the new additional file Fig. S6c. Moreover, we have better explained this part in the text and modified the text of figure 4d.*

*Old text*

*TES and SES are enriched among the hyper-acetylated H3K27 regions in HDAC4-/- cells (Fig. 4d). Hence, we speculated that HDAC4 could directly control the folding of chromatin at TES and SES. ChIP-seq was performed to map HDAC4 genomic binding and explore its regulatory properties on these elements. Fig. 4d shows that HDAC4 binding in WT cells is enriched in correspondence of loci characterized by H3K27ac hyper-acetylation and H3K27me3 demethylation after HDAC4 KO. This evidence suggests a direct involvement of HDAC4 in these epigenetic regulations. Moreover, 13.5% of HDAC4 peaks were found to directly control the chromatin folding in SES (Fig. 4e).*

*New text*

*The previous analysis suggests that hyper-acetylated H3K27 regions in HDAC4-/- cells are enriched for TES and SES. Hence, we hypothesized that HDAC4 could directly control H3K27ac levels at TES and SES. To prove it, we used ChIP-seq to map the genomic regions bound by HDAC4. Fig. 4d shows that HDAC4 binding in WT cells is enriched in correspondence of loci characterized by H3K27ac hyper-acetylation and H3K27me3 de-methylation after HDAC4 KO. This evidence suggests a direct involvement of HDAC4 in these epigenetic regulations, as further confirmed by the heatmap analysis for H3K27ac at SES (Additional file1: Fig. S6c). In summary, 13.5% of HDAC4 peaks colocalize with SES (Fig. 4e).*

5.	Figure 4g – the reduction in acetylation after induction of HDAC4 looks modest. Do the authors have a quantification (such as area under the curve) of this reduction across the SESs regulated by HDAC4?

*We performed the suggested analysis for the SESs showed in figure 4g. These new data have been included in the new supplementary table S3. The quantification of H3K27ac levels in the SES regulated by HDAC4 showed in figure 4g confirms the overall increase in acetylation. Interestingly, HDAC4 seems to exert an action on specific regions of these SES. We have inserted a new text in the result section.*

*Old text*

*By contrast, NFKB consensus characterizes SES not directly bound by HDAC4 (Fig. 4g right panel and additional file 3, Additional file 3: Table S2), as exemplified by the IL1B and CXCL8 loci.*

*New text*

*By contrast, NFKB consensus characterizes SES not directly bound by HDAC4 (Fig. 4g right panel and additional file 3, Additional file 3: Table S2), as exemplified by the IL1B and CXCL8 loci. The quantitative analysis confirmed an augmentation in H3K27 acetylation at these SES upon HDAC4 depletion (Additional file 3: Table S2).*

6.	What was the background chosen for motif search? It would be appropriate to consider a union of H3K27ac peaks rather than whole genome as ChIP-seq data often has GC biases.

*We performed motif discovery on 91 HDAC4-gained SES using these sequences to build, as specified in the Methods section, a 1-order background model to normalize for biased distribution of letters and groups of letters in the analyzed sequences. Specifically, this kind of model adjusts for dimer biases (e.g., GC content in DNA sequences). We did not use whole genome ChIP-seq data and we modeled the background to specifically deal with the kind of concern raised by the Reviewer. Hence, we are confident of having already met in a satisfying way this Reviewer’s concern.*

*In the revised version of the manuscript, we have better explained it in the materials and methods section: Old sentence*

*The following parameters were used: -ccut 0; -order 1; -meme-maxsize 100000000; -mememinsites 2; -meme-maxsites 100; -mememinw 6; -meme-maxw 10; -meme-nmotifs 10; - meme-mod anr; -dreme-e 0.05; -centrimo-score 5.0; -centrimo-ethresh.*

*New sentence*

*The following parameters were used: -ccut 0; -order 1; -meme-maxsize 100000000; -mememinsites 2; -meme-maxsites 100; -mememinw 6; -meme-maxw 10; -meme-nmotifs 10; - meme-mod anr; -dreme-e 0.05; -centrimo-score 5.0; -centrimo-ethresh. Using a 1-order background model to normalize for biased distribution of letters and groups of letters in the analyzed sequences, allows to specifically adjust the background for dimer biases (e.g. GC content).*

7. It is not clear if the ChIP signals throughout the manuscript (for example in Figure 4g, 6c) were normalized to H3. This is very important particularly in senescent cells as they are known to downregulate histones.

*We used the MAnorm method (Shao Z et., Genome biology. 2012 Mar 16;13(3):R16) to compare the ChIP-seq signals in the HDAC4 WT and KO conditions (this latter obtained at 36h after HDAC4 removal), to understand if signal normalization was required. For both H3K27ac/H3K27me3 and H3K4me1 signals, the pre- and post-normalization regression lines were very similar and do not require further normalization. Differently, we normalized H3K27ac levels to total H3 for the qPCR-ChIP data obtained from HDAC4 depleted senescent SK-LMS-1 cells (Fig. 6E) and BJ/RAS cells (in Fig. 6G and 7). Importantly, we recorded a decrease in histone H3 protein levels only starting from 60h after HDAC4 depletion in SK-LMS-1 and from 120h after HRAS G12V expression in BJ cells. For these reasons, we are persuaded that data were analyzed correctly.*

Minor concerns

1.	Please correct typos throughout manuscript, for example “knowledges”, “differentiative” on page 3, “telomers” on page 4, “regulative” on page 7, “knowledges” on page 13 etc.

*Manuscript has been carefully scrutinized for typo errors.*

2.	In the Background section, I would not really mention “SWI/SNF/p300 complex” as they are not a part of the same complex but rather interact with each other. Additionally, the phrase “senescent genetic program” should be replaced. Do the authors mean senescence gene expression program?

*We have followed the Reviewer’s suggestion and better explained the concepts.*

*Old text*

*The balanced action of acetyltransferases (HAT) of the SWI/SNF/p300 complex [11].*

*New text*

*The balanced action of acetyltransferases (HAT) such as p300/CBP, by interacting with the SWI/SNF complex [11].*

*Old text*

*HDAC4 by monitoring H3K27ac levels at selected enhancers and super-enhancers antagonizes the activation of the senescent genetic program.*

*New text*

*HDAC4 by monitoring H3K27ac levels at selected enhancers and super-enhancers antagonizes the activation of the senescence gene expression program.*

3.	Figure 1d-g would benefit from a discussion of why the authors see different effects in different cells used for OIS. For example, why does HDAC9 go up in BJ-TERT cells but decrease in IMR90 cells?

*The regulation of HDAC9 expression is an interesting and still mysterious aspect. In normal tissues it is expressed at very low levels with few exceptions (see also our recent review (Life 2021;11:90). The oncogenic transformation often increases its expression, as shown in figures 1d, e and f, for BJ cells. As noted by the reviewer, HDAC9 levels are reduced in IMR90/TERT/RAS, compared to untransformed cells (Fig. 1g). However, the higher percentage of SA-b-gal positive cells suggests that IMR90-TERT/RAS cells are in a more advanced state of senescence compared to BJ. In the revised version of the manuscript, we have explained this point.*

*Old text*

*The same regulation of class IIa HDACs was confirmed in a second model of AKT1-induced OIS (Fig. 1f) and in IMR90 fibroblasts during RAS-induced OIS (Fig. 1g).*

*New text*

*The same regulation of class IIa HDACs was confirmed in a second model of AKT1-induced OIS (Fig. 1f) and in IMR90 fibroblasts during RAS-induced OIS (Fig. 1g). Curiously, RAS triggers a reduction of HDAC9 levels in IMR90 cells compared to BJ cells. The higher percentage of SAb-gal positive cells suggests that IMR90/TERT/RAS cells are in a more advanced state of senescence compared to BJ/TERT/RAS cells.*

4.	The authors mention on page 6, “As expected, NFKB inhibition only partially recovers the senescent phenotype in terms of SA-13-gal positivity and progression through S-phase (Fig. 3j/k).” Could the authors provide a reference? It might be useful to clarify the difference between p53/pRb inhibition versus NFkB inhibition and their distinct effects on senescence in the text.

*We apologize for the lack of the reference We have inserted it. As expected, NFKB inhibition only partially recovers the senescent phenotype in terms of SA-13-gal positivity and progression through S-phase (Genes Dev. 2011 Oct 15;25(20):2125). We have better explained the effect of p53/pRb inhibition versus NFkB inhibition.*

*Old text*

*The effect of HDAC4 was compared to the switching off of the TP53, NFKB or pRB pathways, well-known regulators of the senescence response [2,27].*

*New text*

*The effect of HDAC4 was compared to the silencing of pRB, TP53 or NFKB pathways, wellknown regulators of the senescence response. They are involved in the control of the cell cycle (pRB), in sensing oncogenic lesions/DNA damage (TP53) and in SASP surveillance (NFKB) [2,27].*

5.	Could the authors clarify what they mean by “Next, ChIP-seq was performed after 36h from HDAC4 depletion” on page 8? Isn’t this ChIP-seq from HDAC4-/- cells?

*The ChIP-seq was performed in HDAC4-/- cells engineered to express a PAM-mutated version of HDAC4 fused to ER. In the presence of 4OHT HDAC4 is expressed. In the revised version of the manuscript, we have better explained it. Next, ChIP-seq was performed in HDAC4-/-HDAC4PAM-ER cells after 36h from the removal of 4-OHT.*

6.	On page 9 – please revise the sentence “Moreover, 13.5% of HDAC4 peaks were found to directly control the chromatin folding in SES (Fig. 4e)”. This data indicates HDAC4 peaks are found in SES but doesn’t imply anything about folding and may be misleading to readers. Please also revise the sentence on line 208 of page 8.

*We agree the Reviewer’s comments and we have already modified it in the answer to point 4. New text*

*In summary, 13.5% of HDAC4 peaks colocalize with SES (Fig. 4e).*

*Old sentence*

*Hence, we speculated that HDAC4 could directly control the folding of chromatin at TES and SES.*

*New text*

*Hence, we hypothesized that HDAC4 could directly control H3K27ac levels at TES and SES.*

7.	Please describe the experimental details of AP1 transcriptional activity (Figure 6b) in greater detail in the Methods.

*In the M&M revised version of the manuscript we have inserted the following sentences: For luciferase assay, SK-LMS-1 cells HDAC4 wt or KO re-expressing HDAC4 mutants or HDAC3 were transfected with 300 ng pGL3-3XAp1-luc plasmid (Addgene # 40342) and 30ng pTKRenilla Luc plasmid. After 48h, cells were harvested and the assay was performed accordingly to Dual-Glo Luciferase Assay System (Promega). Luminescence was recorder by using Modulus Luminometer Microplate reader (Turner BioSystems) and Luciferase signal was normalized to Renilla signal.*

8.	dCas9 ChIP should be normalized to input not H3.

*As the reviewer suggested, we normalized respect to the input and the results are identical to the normalization respect to H3. Therefore, within the time-frame of this experiment, we can exclude major variations in H3. We have included the new analysis in this rebuttal.*

*Figure R1. ChIP-qPCR signals using the anti-Cas9 antibody for the indicated loci in BJ/hTERT/RAS cells transfected with the indicated dCas9 chimeras and IVT sgRNAs. The genomic region investigated is indicated with the closest-associated gene.*

*We have inserted a new sentence in the text to clarify this point.*

*When normalization was calculated respect to input the results were identical, thus indicating that, within the time-frame of the experiment, H3 levels remain unperturbed.*

Reviewer #2:

The data is of high quality and the presentation is very clear as well.

*We thank the Reviewer for this comment.*

I have a few comments for the authors to consider in revising the manuscript:

1)	Phosphorylation is a major regulatory event for these enzymes, so I would like to see some data in this regard.

*Phosphorylation is a major regulatory event for these enzymes. Therefore, we have followed the Reviewer’s suggestion and analyzed whether the entrance in senescence is coupled to changes in HDAC4 phosphorylation level. We investigated the phosphorylation status of serine 246 that is important for 14-3-3 binding and nuclear export, using a specific antibody. Since the antibody recognizes also the corresponding serine 259 in HDAC5, we cannot discriminate between the two family members. Interestingly, HDAC4/5 are rapidly dephosphorylated after RAS induction. Moreover, in BJ/TERT, HDAC4 shows a consistent nuclear localization that is largely unperturbed after 2 day of RAS induction, once serine 246 dephosphorylation is massive. In summary, although the result on serine 246 phosphorylation is very clear, its implications on HDAC4 nuclear/cytoplasmic shuttling in this context are mysterious.*

*In the revised version of the manuscript, we have included new supplementary figures and added a new text describing these new data in the result section.*

*Old text*

*Similarly, in senescent cells the treatment with LiCl (Additional file 1: Fig. S1i) or the silencing*

*of GSKJ3 are both capable of restoring HDAC4 levels (Fig. 1j).*

*New text*

*Similarly, in senescent cells the treatment with LiCl (Additional file 1: Fig. S1i) or the silencing of GSKJ3 are both capable of restoring HDAC4 levels (Fig. 1j).*

*Class IIa HDACs nuclear/cytoplasmic shuttling is intensively regulated by phosphorylation. We investigated the levels of serines 246/259 of phosphorylation, found respectively in HDAC4 and HDAC5, during RAS-induced senescence (RIS). These serines, that are binding sites for 14¬3-3 proteins and involved in the nuclear export, are rapidly dephosphorylated upon RAS induction (Additional file 1: Fig. S1j). However, HDAC4 shows a pan/nuclear localization in BJ-TERT cells and this localization is largely unperturbed after RAS activation (Additional file 1: Fig. S1k). In conclusion, dephosphorylation at serines 246/259 is an early response to RAS induction coupled to the proliferative boost.*

2)	MEF2 is a key DNA-binding partner, whereas c-Jun/Fos is not so well established. On the other hand, MEF2 may regulate the expression c-Jun/Fos, so I would suggest to analyze whether the impact on H3K27 modifications are direct.

*The Reviewer is right. MEF2 can regulate the expression AP-1 members. Recently, we have confirmed that MEF2 can control JUN expression in SK-LMS-1 cells (PLoS Genet. 2017 Apr 18;13(4):e1006752). We have analyzed JUN expression during senescence induced by HDAC4 ablation in a time-course experiment. JUN expression is transiently upregulated at early times from HDAC4 removal and later on, during the establishment of senescence (see the figure below).*

*In the revision version of the manuscript, we have inserted a new supplementary figure S6e showing this result.*

*Additional file 1: Figure S6e. Regulation of JUN expression by HDAC4.*

*JUN mRNA expression levels in SK-LMS-1 HDAC4-/- cells, re-expressing (+4OHT) or not (-4OHT) HDAC4PAM-ER. The levels are relative SK-LMS-1 HDAC4-/-HDAC4PAM-ER grown in the presence of 4OHT. Mean ± SD; n = 3.*

*Accordingly, we have inserted a new text in the discussion*

*Old text*

*During senescence entrance, the buffering activity of HDAC4 is lost, as a consequence of its controlled UPS-mediated and GSK3β-assisted degradation, thus TS and SE can orchestrate the transcriptional re-programming.*

*New text*

*During senescence entrance, as a consequence of the GSK3β-assisted degradation, the buffering activity of HDAC4 is lost and TS/SES can orchestrate the transcriptional re-programming. Certainly, the mechanisms engaged by HDAC4 to silence these regions deserve further characterizations and there are several possibilities. HDAC4 could, directly or indirectly, repress AP-1 mediated transcription or, through MEF2, it could also control JUN levels (PLoS Genet. 2017 Apr 18;13(4):e1006752.) (Additional file 1: Fig. 6e).*

3) There are reports that HDAC4 recruits HDAC3 for deacetylation and that HDAC3 is known to deacetylase H3K27, so the authors may need to sort this out as well.

*The Reviewer is right aging. HDAC4 can recruit HDAC3. For this reason, we investigated the contribution of HDAC3. In figure 6e we show that two super-enhancers characterized by HDAC4 binding (AKR1E2 and VEGFC) are marked by HDAC3 binding too. In this super-enhancers HDAC4 as well as HDAC3 can control H3K27 acetylation. HDAC3 is particularly active on VEGFC SE. In these two examples HDAC3 seems to act also independently from HDAC4. In a second experiment, we used dCas9 to load the two HDACs at the SE of different genes (IL1B, SOD2, CCXL8 and AREG). When we forced HDAC3 to bind some of these enhancers, it can reduce H3K27ac levels at IL1B, CCXL8 and SOD2 SEs but not at AREG SE.*

*Whereas HDAC4 was efficient in reducing H3K27ac at AREG SE. It is evident that these preliminary results need further confirmation, however they suggest that HDAC4 can also exert HDAC3-independent activities. Certainly, new experiments are necessary to fully explain the contribution of HDAC3 in the HDAC4 context. In this perspective it will be important to compare the genome binding ability of HDAC3 in the presence and absence of HDAC4. It is a study that we have in plan for the future.*

*In the revised version of the manuscript, we have better emphasized this point in the discussion.*

*Old text*

*Especially if the repressive effect is compared to its “natural” SES (AREG and SOD2).*

*Therefore, some SES and TES could be indirectly controlled by HDAC4 in hierachical manner.*

*New text.*

*Especially if the repressive effect is compared to its “natural” SES (AREG and SOD2). Therefore, some SES and TES could be indirectly controlled by HDAC4 in a hierachical manner. In general, HDAC4 and class IIa HDACs can form complexes with class I HDACs and with HDAC3 in particular (Epigenomics. 2016 Feb;8(2):251-69). We have observed that H3K27ac level at some SE can be modulated by both HDAC4 and HDAC3 whereas, at AREG SE, H3K27ac is modulated only by HDAC4. These preliminary observations deserve further investigations to precisely map the genomic regions bound by these two epigenetic regulators.*

4) For immunoblotting mouse aging (Fig. 1C), I would suggest to add results on muscle and brain as HDAC4 is much more important in those tissues.

*We do not have currently access to aged brain tissues. Nevertheless, HDAC4 downregulation in the senescence-accelerated mouse (SAM) brain, an accelerated model of aging (Exp Gerontol. 2005; 40:774-83) was previously described (Clin Exp Pharmacol Physiol. 2016; 43:41-6).*

*In the revised version of the manuscript, we have inserted a new sentence highlighting this point.*

*Old text*

*As expected, aged tissues are characterized by high TP53 levels and Lamin-B1 downregulation*

*(Fig. 1c).*

*New text*

*As expected, aged tissues are characterized by high TP53 levels and Lamin-B1 downregulation (Fig. 1c). In agreement with our data, in mice representing a senescence-accelerated model of aging, the downregulation of HDAC4 was previously observed (Clin Exp Pharmacol Physiol. 2016; 43:41-6).*

*Concerning the aging in skeletal muscle tissue, the question is more complex. In addition to cellular senescence aged muscle are characterized by atrophy and by alterations in neuro¬muscular junction formation and maintenance (J Physiol. 2017; 595:6409–6415). The last condition could trigger the upregulation of HDAC4 (Mol. Biol. Cell. 2009; 20:1120–1131) and of HDAC9 (Nat. Neurosci. 2005;8:313–321). We have investigated class IIa HDACs levels in slow-twitch soleus and in the mixed gastrocnemius. A downregulation of HDAC4 can be appreciated in both muscle types from older mice. HDAC9 seems to be expressed at very low levels only in the soleus and is downregulated in older mice. HDAC7 can be detected only in the soleus, without evident expressional changes with aging. It is possible that, the specific HDAC7 positivity of the soleus, stems from the high percentage of vascular tissue.*

*When we evaluated Tp53 levels, different from skin and liver its expression is not increased with aging. Since these results are preliminary and also considering the specific regulation of class IIa HDAC in skeletal muscle, although they strengthened the concept of HDAC4 downregulation with aging, we prefer to omit them from the manuscript. We have prepared a figure for the reviewer that includes the data (see below).*

*Figure R1. Class IIa HDACs expression in muscle tissue during aging.*

*A)	Immunoblot analysis in tissue-derived lysates obtained from male mice sacrificed at 196 (young) and 720 (old) days of age using the indicated antibodies. Actin was used as loading control.*

*B)	mRNA expression levels of the indicated genes in young (196 days) and old (270 days) mice, as analyzed by qRT-PCR. Mean ± SD; n = 3.*

5) The title is a bit long and could be shortened to make it more catchy. Also, the word 'anticipate" on page 5 should be replaced with words such as "promote", "stimulate" and "enhance."

*We agree that the title is a bit long. However, it is self-explanatory. In the revised version of the manuscript, we have shortened it a little bit.*

*Old title*

*HDAC4 is degraded during senescence entrance to unleash an epigenetic program driven by*

*AP1/p300 at selected enhancers and super-enhancers*

*New title*

*HDAC4 degradation during senescence unleashes an epigenetic program driven by AP1/p300*

*at selected enhancers and super-enhancers*

**Second round of review**

**Reviewer 1**

Response to point #1: The question remains whether the authors failed to observe a decrease of HDAC4 in male mice tissues. The authors should clearly state the sex-specificity of their observation (if they have done the experiments) or the fact that it is yet to be tested.

Response to point #4: Ok. And I would add in the new text, that the reasons for H3K27me3 increase in some loci remain to be tested. If they are not at the same loci, I am not sure the change in H3K27me3 adds anything meaningful to this paper.

Response to point #5: I apologize that I mentioned “reduction in acetylation after induction of HDAC4”, I meant increase in acetylation after depletion of HDAC4.

I am afraid I cannot see Supp table 3. What I meant was to quantify across all SESs bound by HDAC4, not just the loci in Fig. 4g. Please represent this as a metaplot across all SESs.

Response to point #7: Ok. This information needs to be included in the Methods Section.

**Authors Response**

**Point-by-point responses to the reviewers’ comments:**

1. The question remains whether the authors failed to observe a decrease of HDAC4 in male mice tissues. The authors should clearly state the sex-specificity of their observation (if they have done the experiments) or the fact that it is yet to be tested.

*In the revised version of the manuscript, we have inserted the following sentence:*

*In humans, female longevity tends to exceed male longevity, for this reason we investigated Hdac4 levels specifically in female mice (Psychol Health Med. 2015;20:129).*

Ok. And I would add in the new text, that the reasons for H3K27me3 increase in some loci remain to be tested. If they are not at the same loci, I am not sure the change in H3K27me3 adds anything meaningful to this paper.

*We have inserted the new text as the Reviewer suggest.*

*The reasons for H3K27me3 increase in some loci remain to be tested.*

*We believe that the characterization of H3K27me3 is informative of the wider effects of HDAC4 on the chromatin status and for this reason we prefer to keep these data in the manuscript.*

I am afraid I cannot see Supp table 3. What I meant was to quantify across all SESs bound by HDAC4, not just the loci in Fig. 4g. Please represent this as a metaplot across all SESs.

*We have verified the download of the supplementary file 4: table S3 and it works.*

*In this revised version as requested, we have analyzed all SES bound by HDAC4 for variations in H3K27ac levels between WT and KO cells. We performed this analysis using the SES defined in SK-LMS-1 cells, by the ROSE algorithm. These SE are not detected in growing SK-LMS-1 cells expressing HDAC4. It is possible that this list includes SE that are modulated at early times during the senescent progression. This new analysis has been included in the new additional figure S6e.*

*Figure S6. HDAC4 supervises enhancers and super-enhancers activated early during senescence.*

*a.	Heat-map of the 139200 H3K27me3 enriched peaks (“GLOBAL”) and of the associated H3K27ac signal in the same regions in the indicated SK-LMS-1 cells. The analysis was performed in a region of ±15Kb around peak summit.*

*b.	Heat-map of the 1205 H3K27me3 peaks (“FC>2”) displaying an overall signal ratio (WT/KO)>2 and of the associated H3K27ac signal in the same regions. The analysis was performed in a region of ±15Kb around peak summit.*

*c.	Heat-map of the HDAC4 (WT), H3K27ac (KO), and H3K27me3 (WT) signals in the 91 SES containing HDAC4 enriched peaks. The analysis was performed in a region of ±20Kb around peak summit.*

*d.	Detailed view of the H3K27ac and H3K27me3 levels in the CDKN1A locus in WT and HDAC4 KO SK-LMS-1 cells.*

*e.	Heat-map of the HDAC4 (WT), H3K27ac (KO), and H3K27ac (WT) signals in SES of SK-LMS-1 cells containing HDAC4 enriched peaks. The analysis was performed in a region of ±10Kb around peak summit. The ROSE algorithm was used to separate super-enhancers from typical enhancers. SE defined in SK-LMS-1/HDAC4-/- cells expressing the tamoxifen inducible version of HDAC4 (WT) were subtracted from SE defined in the same cells grown for 36h without 4-OHT. The obtained 363 SE represent the SES of SK-LMS-1 cells. These SES were next selected for the presence of HDAC4 peaks (n=27).*

*f. JUN mRNA expression levels in SK-LMS-1 HDAC4-/- cells, re-expressing (+4-OHT) or not (-4-OHT) HDAC4PAM-ER. The levels are relative to SK-LMS-1 HDAC4-/-HDAC4PAM-ER grown in the presence of 4OHT. Mean ± SD; n = 3.*

*In the result section we have introduced the following sentence*

*Old text*

*Using the ROSE algorithm 980 SE modulated by HDAC4 were identified (Additional file 5: Table S4). 39% of them were previously defined as SES in other cell lineages (Fig. 5a). Relying on the importance of HDAC4*

*New text*

*Using the ROSE algorithm 980 SE were globally identified in HDAC4-/- cells (Additional file 5: Table S4). 39% of them were previously defined as SES in other cell lineages (Fig. 5a) and 363 were specific of HDAC4-/- cells. This group of SE represents the SE induced during the early phases of senescence (36 hours from HDAC4 depletion) in SK-LMS-1 cells. 27 of these SES are marked by HDAC4 binding. The heat maps and the metaplots for H3K27ac levels in these SES are illustrated in the Additional file 1: figure S6e. Relying on the importance of HDAC4*

Ok. This information needs to be included in the Methods Section.

*In the revised version of the manuscript, we have inserted this information in the Methods section.*

*We used the MAnorm method (Shao Z et., Genome biology. 2012 Mar 16;13(3):R16) to compare the ChIP-seq signals in the HDAC4 WT and KO conditions. For H3K27ac, H3K27me3 and H3K4me1 signals, the pre- and post-normalization regression lines were very similar and did not require further normalization. For the qPCR-ChIP experiments, normalization was against total H3. Under the time of the analysis H3 protein levels were not decreased. A decrease in H3 was observed only at much later times after HDAC4 depletion.*
